# Gene co-expression network analysis identifies porcine genes associated with variation in *Salmonella* shedding

**DOI:** 10.1186/1471-2164-15-452

**Published:** 2014-06-09

**Authors:** Arun Kommadath, Hua Bao, Adriano S Arantes, Graham S Plastow, Christopher K Tuggle, Shawn MD Bearson, Le Luo Guan, Paul Stothard

**Affiliations:** Department of Agricultural, Food and Nutritional Science, University of Alberta, Edmonton, AB Canada; Department of Animal Science, Iowa State University, Ames, IA USA; USDA/ARS/National Animal Disease Center, Ames, IA USA

## Abstract

**Background:**

*Salmonella enterica* serovar Typhimurium is a gram-negative bacterium that can colonise the gut of humans and several species of food producing farm animals to cause enteric or septicaemic salmonellosis. While many studies have looked into the host genetic response to *Salmonella* infection, relatively few have used correlation of shedding traits with gene expression patterns to identify genes whose variable expression among different individuals may be associated with differences in *Salmonella* clearance and resistance. Here, we aimed to identify porcine genes and gene co-expression networks that differentiate distinct responses to *Salmonella* challenge with respect to faecal *Salmonella* shedding.

**Results:**

Peripheral blood transcriptome profiles from 16 pigs belonging to extremes of the trait of faecal *Salmonella* shedding counts recorded up to 20 days post-inoculation (low shedders (LS), n = 8; persistent shedders (PS), n = 8) were generated using RNA-sequencing from samples collected just before (day 0) and two days after (day 2) *Salmonella* inoculation. Weighted gene co-expression network analysis (WGCNA) of day 0 samples identified four modules of co-expressed genes significantly correlated with *Salmonella* shedding counts upon future challenge. Two of those modules consisted largely of innate immunity related genes, many of which were significantly up-regulated at day 2 post-inoculation. The connectivity at both days and the mean gene-wise expression levels at day 0 of the genes within these modules were higher in networks constructed using LS samples alone than those using PS alone. Genes within these modules include those previously reported to be involved in *Salmonella* resistance such as *SLC11A1* (formerly *NRAMP1*), *TLR4*, *CD14* and *CCR1* and those for which an association with *Salmonella* is novel, for example, *SIGLEC5*, *IGSF6* and *TNFSF13B*.

**Conclusions:**

Our analysis integrates gene co-expression network analysis, gene-trait correlations and differential expression to provide new candidate regulators of *Salmonella* shedding in pigs. The comparatively higher expression (also confirmed in an independent dataset) and the significantly higher connectivity of genes within the *Salmonella* shedding associated modules in LS compared to PS even before *Salmonella* challenge may be factors that contribute to the decreased faecal *Salmonella* shedding observed in LS following challenge.

**Electronic supplementary material:**

The online version of this article (doi:10.1186/1471-2164-15-452) contains supplementary material, which is available to authorized users.

## Background

*Salmonella enterica* serovar Typhimurium is a gram-negative zoonotic bacterium that can colonise the gut of humans and many species of food producing farm animals and cause enteric or septicaemic salmonellosis [1]. In pigs, infections by *S.* Typhimurium mostly lead to a localised enterocolitis, which is responsible for significant economic losses to the pig industry [2]. An unknown percentage of *Salmonella* infected pigs continue to be asymptomatic carriers even after acute response, thereby posing long-term zoonotic threats through contaminating the pork production chain. Prevention and control of salmonellosis in pigs thus assumes great importance not only for animal welfare, reduced antibiotic use and improved profitability of pig industry but also for minimizing risks to public health [3].

Host genetic response to *Salmonella* infection has been well studied in several species. A review by Roy and Malo [4] has reported several genes to be involved in regulation of responses to *Salmonella* infection in mice, for example, *SLC11A1* (formerly *NRAMP1*), *TLR4*, *BTK*, *LBP*, *CD14*, *CYBB*, *NOS2*, *TNF*, *IL12*, *IFNG*, *IL12B*, *TLR5* and others. Clinical manifestations associated with *Salmonella* infection are dependent on several factors such as the serotype of *Salmonella* involved, host species affected and age of the host. While infection with *S.* Typhimurium induces a systemic disease similar to human typhoid fever in mice, the infection is mostly of the enteric form in pigs, except in the case of very young piglets [1]. Thus a different set of genes may contribute to resistance against *Salmonella* infection depending on the host species and the *Salmonella* serotype involved. Indeed, studies of *Salmonella* resistance in chicken report different genes depending on whether the infection is systemic or enteric [5]. For example, the *SLC11A1* gene and the *SAL1* locus confer resistance to systemic Salmonellosis [6, 7], whereas several members of the gallinacin gene family confer resistance to enteric Salmonellosis [8].

Studies on several species have shown that host genetic variations in natural populations contribute to varying responses to different pathogens in terms of resistance or increased susceptibility [9–11]. Distinct responses to *Salmonella* infection have been observed in pigs, some recovering faster and shedding lower levels of *Salmonella* in faeces than others (low shedders, LS versus persistent shedders, PS) [12]. This trait variation could indicate the existence of a genetic component to *Salmonella* shedding and resistance that may be exploited in animal breeding and disease diagnostics. Uthe et al. [13] reported SNPs in ten genes, including *GNG3*, *NCF2* and *CCR1*, that were associated with *Salmonella* shedding in pigs. While many studies have looked into the host genetic response to *Salmonella* infection, relatively few have used trait-gene expression correlation to identify genes whose variable expression among uninfected individuals may be associated with differences in *Salmonella* clearance and resistance. For example, several genes involved in innate immunity (*IL8*, *IL18*, *IFNG*, *TLR4*, *iNOS*, *GAL1*, *GAL2*) have been shown to be either constitutively or inductively more highly expressed in caecal tonsils of *Salmonella* resistant strains of chicken compared to susceptible strains [14]. A study on *Mycobacterium tuberculosis* infection in humans identified a SNP that appears to control susceptibility to tuberculosis through its effects on the expression of the *DUSP14* gene in dendritic cells prior to infection [15]. Differences in gene expression prior to Porcine Reproductive and Respiratory Syndrome (PRRS) virus infection have been observed among pigs belonging to different phenotypic groups as defined by the viral load and weight gain of individual pigs observed during a defined number of days following infection [16]. Such differences in gene expression among different phenotypic groups have been attributed to the differences in the genetic background of individual pigs [17]. Taken together, the findings reported above may indicate that genetic resistance to infections is mediated in part through the presence of a more activated defense system in resistant individuals compared to susceptible individuals so that, in the event of an infection, resistant individuals can mount a faster and more effective immune response.

In this study we assess the whole blood transcriptome prior to and following infection. Whole blood consists of cells that form integral parts of the immune system and whole blood can be easily and repeatedly sampled. The whole blood transcriptome provides a ‘snap shot’ of the complex immune networks that operate throughout the body [18] and several studies in humans and animals have analysed the blood transcriptome to provide new insights into host immune responses against a wide variety of pathogens and to identify potential biomarkers [16, 19, 20].

A recent microarray based study [20] identified several hundreds of differentially expressed (DE) genes including *SLC11A1* and *TLR4* when comparing the whole blood transcriptome of pigs before (day 0) and two days after *Salmonella* inoculation (day 2) and also reported significant differences in the number and expression profiles of DE genes post inoculation in LS compared to PS. However, the DE analysis performed in those studies failed to identify significant differences in expression of genes between LS and PS before *Salmonella* challenge that could potentially explain or predict the varying responses between the two groups upon infection. This absence of DE could be due to the fact that the differences in expression of host resistance genes against *Salmonella* between LS and PS may be subtle, unlike the gene expression differences between non-infected and infected states, and thus requires a broader or more sensitive approach to identify those genes.

DE analysis typically focuses on identifying genes with the most significant differences between contrasting conditions. In addition, the requirement for multiple testing corrections may further impede the discovery of genes with subtle differences in expression. A powerful alternative approach for gene expression analysis is co-expression analysis, which considers the relationships between measured transcripts in an unsupervised way. The weighted gene co-expression network analysis (WGCNA) approach [21], one of the popular methods developed for gene co-expression analysis, effectively overcomes the multiple testing problems inherent in high throughput gene expression data analysis. This methodology begins with the identification of modules of highly correlated genes assessed by pair-wise correlations between gene expression profiles. Next the relationships between only a few tens of modules and the phenotypic trait of interest are considered. Finally candidate genes associated with the trait are prioritised based on network statistics like module membership and gene significance. WGCNA has been used to identify genes and gene networks associated with specific tissues, distinct biological states or diseases, and qualitative as well as quantitative phenotypes [22–24].

Here, we aimed to identify porcine genes and gene co-expression networks that differentiate distinct responses to *Salmonella* challenge with respect to faecal *Salmonella* shedding, using WGCNA and RNA-Seq data from whole blood.

## Results

### Faecal *Salmonella* shedding counts

The pigs used in this study were identified as LS or PS based on the cumulative area under the plotted log curve (AULC) of their faecal *Salmonella* shedding counts (see Methods) (Table 1). For LS, the AULC ranged from 29.3 to 86.6 (mean = 67.9 ± 17.4) whereas for PS, it ranged from 133.2 to 186.5 (mean = 159 ± 18.4). The lower the AULC, the lower the faecal shedding counts estimated for that pig. We predict that a lower early shedding count will accelerate the return of the animal to a healthy (non-shedding) status following *Salmonella* challenge.

**Table 1:** **Shedding status of pigs determined based on AULC of faecal** ***Salmonella*** **shedding counts**

Low shedders		Persistent shedders	
Pig ID	AULC *	Pig ID	AULC *
138	29.3	125	133.2
116	66.3	70	143.6
144	67.2	83	144.8
109	68.1	136	155.9
18	70.2	141	160.2
73	70.9	4	174.5
82	84.3	30	174.8
39	86.6	28	186.5

*AULC: cumulative area under the plotted log curve of the logarithmically normalised faecal *Salmonella* shedding counts obtained between day 0 to day 20 post-inoculation for each individual.

### RNA-Seq profiling of porcine whole blood expressed genes and their functional classification

RNA extracted from porcine peripheral blood samples collected at day 0 and day 2 post inoculation (p.i.) of *Salmonella* from 16 selected pigs identified as LS (n = 8) and PS (n = 8) were depleted for globin transcripts and subjected to Illumina sequencing. The sequencing depths achieved for these samples ranged from 23 to 52 million reads (mean = 37 ± 6), of which approximately 90% (mean = 33 ± 6) mapped to the pig genome (Additional file 1), identifying a total of 21,638 expressed genes. The efficiency of the globin depletion protocol varied across samples with the total globin reads (*HBA* + *HBB*) ranging from 0.2-60% of the total mapped reads post-depletion (Additional file 2). Based on data from a different set of samples (n = 12) that were used to develop the globin depletion protocol [25] (manuscript under preparation), it is known that the total globin reads constitute 26 to 54% (average 46.1 ± 8.6%) of the total mapped reads in blood of pigs between 3–7 weeks of age. Using this information on normal globin levels in pig blood, we estimate the globin depletion protocol to have worked well for 16 of the 32 samples used here where the total globin reads constituted below 15% of the total mapped reads. For 7 of the 32 samples, the protocol seemed to have worked to some extent, limiting total globin reads to between 20-30% of the total mapped reads. For the remaining 9 samples, however, the protocol either worked poorly (2 samples with total globins between 35-40%) or did not work at all (7 samples with >40% total globins). The differing efficiencies of globin depletion would have affected our ability to detect genes expressed at very low levels via improved coverage for non-globin genes. However, this would not affect the analysis we do here as we filter out the genes expressed at very low levels across all samples. The globin genes were also removed from the gene expression matrix prior to normalisation and further analysis. Removal of genes expressed at very low levels (keeping only genes with counts per million (CPM) per sample > 1 in at least 8 samples) resulted in a set of 10,872 genes expressed in porcine peripheral blood. A multi-dimensional scaling in two dimensions (see Methods) performed using this set of expressed genes revealed a clear separation of the samples by day (day 0 vs. day 2 p.i.) but not by shedding status (LS vs. PS) on either day (Additional file 3).

For a broad overview of the functions attributed to the genes expressed in porcine blood, we performed a functional classification based on gene ontology (GO) terms from GO Slim using the PANTHER gene list analysis tool (see Methods). In the biological processes category (Additional file 4), 26.3% of the blood-expressed genes were annotated to metabolic process, 16% to cellular process, 10.2% to cell communication and 7.7% to transport. In the molecular functions category, 33.8% of the genes were annotated to binding and 30.8% to catalytic activity whereas in the cellular composition category, the dominant term was intracellular (59.9%).

### Gene co-expression network constructed using gene expression data from uninfected pigs

**Figure 1:**
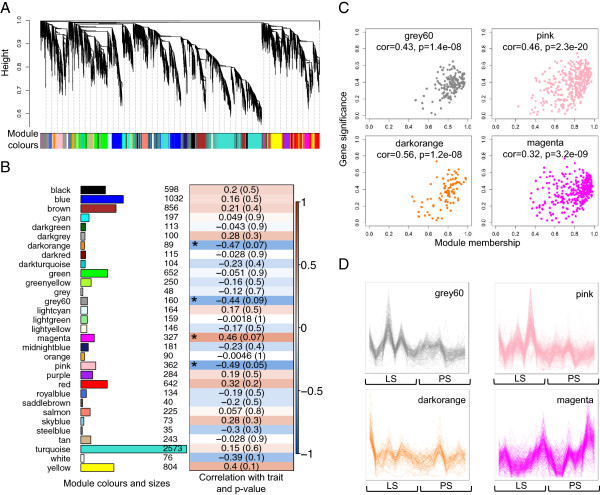
**Module identification in day 0 samples and correlation of module expression with** ***Salmonella*** **shedding counts. A**. Clustering dendrogram of genes showing module membership in colours. **B**. Module sizes and correlation of module eigengenes with trait, AULC of faecal *Salmonella* shedding counts. The module-wise correlations are provided along with the p-value of the correlation in cells coloured by the strength of the correlation. Modules significantly associated with the trait (absolute correlation greater than 0.3 and p-value less than 0.1) are indicated with an asterisk (*). **C**. Scatterplots of gene significance versus module membership for *Salmonella* shedding associated modules, along with correlations and p-values indicated. **D**. Gene co-expression patterns within *Salmonella* shedding associated modules. The samples are sorted from low to high based on shedding counts.

Gene expression profiles of day 0 samples of pigs were analysed using WGCNA in order to identify gene co-expression patterns that may be associated with differences in faecal *Salmonella* shedding counts of those pigs upon future challenge. After setting a minimum module size of 30 and merging modules with highly correlated (*r* > 0.85) eigengenes (defined as the first principal component of a given module and may be considered as a representative of the gene expression profiles in that module), a total of 30 modules were found (excluding the grey module, which is used to hold all genes that do not clearly belong to any other module). These modules will be referred to by their colour labels henceforth, as is standard practice. The modules labelled by colours are depicted in the hierarchical clustering dendrogram provided in Figure 1A. To evaluate the robustness of the identified modules, we performed module stability analysis from bootstrapped networks (see Methods). The module representations were fairly consistent across the majority of the bootstrapped networks (Additional file 5).

### Modules associated with differences in faecal *Salmonella* shedding

To determine if any of the 30 modules of co-expressed genes identified in day 0 samples were associated with the observed differences in faecal *Salmonella* shedding counts upon future *Salmonella* challenge, we tested the correlations of the module eigengenes with the trait, AULC of faecal *Salmonella* shedding counts. Four modules were found significant at the defined cut-offs (absolute correlation > 0.3 and p-value < 0.1). Of these, the *pink*, *grey60* and *darkorange* modules showed negative correlations with the trait while the *magenta* module showed a positive correlation. Figure 1B depicts the module sizes and correlations of the module eigengenes to the trait. The module membership (MM) versus gene significance (GS) plots for these modules (Figure 1C) showed that MM and GS are highly correlated, indicating that genes most significantly associated with the trait are often also the most important (central) elements of the respective modules. Here, MM is a measure of the strength of a particular gene’s membership in a module obtained by correlating the gene’s expression profile with the module eigengene of that module. Highly connected intramodular hub genes tend to have high MM values to the respective module. GS is a measure of the biological relevance of a gene with respect to the trait of interest obtained by correlating the gene’s expression profile with the trait. The gene expression profiles of genes within the four modules associated with *Salmonella* shedding, across all samples ordered by AULC of *Salmonella* shedding counts (Figure 1D), indicate tight co-regulation with an overall higher expression in LS than PS for the *pink*, *grey60* and *darkorange* modules and a lower expression in LS than PS for the *magenta* module. Gene ontology enrichment tests (see Methods) revealed that the *grey60* and *pink* modules were related to immune functions whereas the *darkorange* module was related to signalling processes (Benjamini-Hochberg corrected p-value < 0.05). The positively correlated *magenta* module was not significantly enriched for any GO term. The Ensembl IDs of the genes within these four modules are provided in Additional file 6 and the complete lists of GO terms enriched in these modules are provided in Additional file 7.

### *Salmonella* shedding associated modules identified at day 0 are largely preserved at day 2

**Figure 2:**
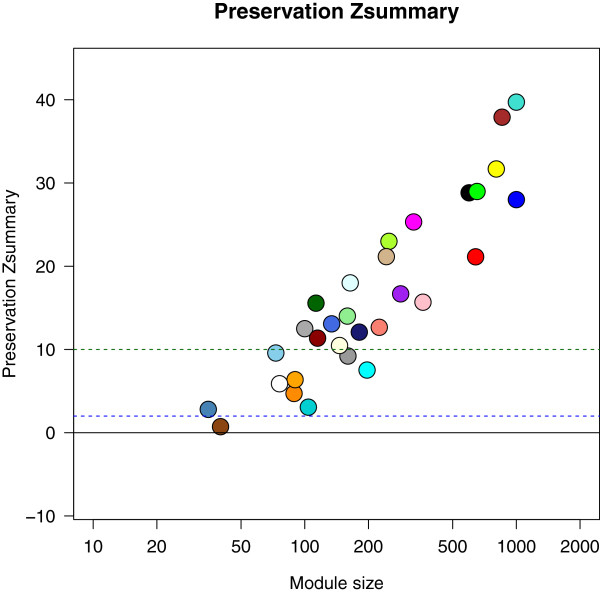
**Module preservation between day 0 and day 2 modules.** Coloured circles denote the Zsummary preservation scores of day 0 modules with day 2 modules. A *Zsummary* score below 2 indicates no preservation, a score above 10 indicates strong preservation and a score between 2 to 10 indicates weak to moderate evidence of preservation.

We speculated that the *Salmonella* shedding associated modules found at day 0 may be preserved at day 2 and have a majority of their constituent genes still co-expressed if they indeed are involved in response to *Salmonella* challenge. Therefore, we first constructed a co-expression network using day 2 samples and tested for module preservation between day 0 and day 2 using the *modulePreservation* method [26] implemented in WGCNA package that calculates *Zsummary* preservation scores. A *Zsummary* score below 2 indicates no preservation, a score above 10 indicates strong preservation and a score between 2 to 10 indicates weak to moderate evidence of preservation [26]. Most of the day 0 modules (21 of 30 modules) were seen to be strongly preserved at day 2 including the *pink* and *magenta* modules (Figure 2), eight were weakly to moderately preserved including the *grey60* and *darkorange* modules and only one was not preserved.

### Differences in expression levels of *Salmonella* shedding associated genes between low and persistent shedders and between days

**Figure 3:**
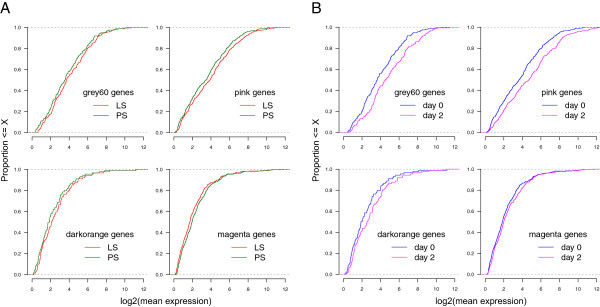
**Differential expression of genes within** ***Salmonella*** **shedding associated modules.** Expression levels of genes within *Salmonella* shedding associated modules in low and persistent shedders at day 0 **(A)**, and in day 0 and day 2 samples **(B)**. The x-axis refers to the log2 transformed mean expression of genes within a particular module in the LS and PS samples **(A)** or in the day 0 and day 2 samples **(B)**. The y-axis refers to the proportion of those genes whose log2 transformed mean expression falls below a particular expression level.

**Figure 4:**
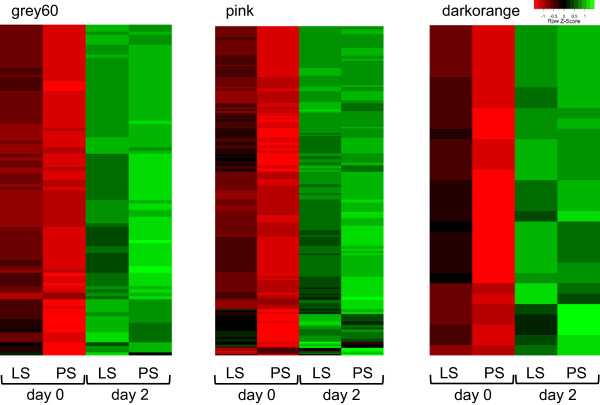
**Expression profiles of selected genes from the** ***Salmonella*** **shedding associated modules that were also up-regulated post-inoculation with** ***Salmonella.*** Heat maps depicting the mean expression profiles of genes within *Salmonella* shedding associated modules (except *magenta*) that were up-regulated post-inoculation. In general, these genes had a higher mean expression in LS than PS at day 0. A majority of the genes in *magenta* module were not differentially expressed between days and thus the association of this module with *Salmonella* shedding counts may not be biologically relevant.

DE analyses between day 0 and day 2 performed separately for LS and PS samples identified 951 genes DE (752 up-regulated and 199 down-regulated) in LS and 2439 genes DE (1400 up-regulated and 1039 down-regulated) in PS (corrected p < 0.05, fold-change > 1.5, CPM > 4), with an overlap of 867 (719 up-regulated and 148 down-regulated) genes. However, only 25 genes were significantly DE between LS and PS at day 2. The low number of genes DE between LS and PS at day 2 was due to the fact that the direction of change was similar for most genes in both groups, though the degree of change in expression between days were higher in PS. From the expression patterns of the modules of co-expressed genes at day 0 that were associated with differences in faecal *Salmonella* shedding counts, it was observed that genes within the *grey60*, *pink* and *darkorange* modules were, in general, more highly expressed in LS than PS at day 0 whereas the opposite trend was observed for genes within the *magenta* module (Figure 3A). If the genes within the modules are influencing *Salmonella* shedding and thereby pathogen clearance and host resistance, one might hypothesise that genes within the modules negatively associated with *Salmonella* shedding at day 0 would have a general up-regulation post-inoculation and those within the positively associated module would have a general down-regulation. To test this hypothesis, we first determined genes differentially expressed (DE) between day 0 and day 2 p.i. using samples from both LS and PS taken together. A total of 1893 genes were found DE (corrected p < 0.05, fold-change > 1.5, CPM > 4) of which 1171 were up-regulated and 722 down-regulated at day 2 p.i. (heat maps in Additional file 8). Approximately 60% of the genes in both the *pink* (213 of 362) and *grey60* (100 of 160) modules were up-regulated at day 2 p.i and 7 and 3 genes were down-regulated in the two modules, respectively. For genes in the *darkorange* module, 36% (32 of 89) were found significantly up-regulated at day 2 p.i. and none down-regulated. Even when considering all genes in the three modules (significantly DE and non-DE), the majority were more highly expressed at day 2 p.i. compared to day 0 (Figure 3B). Thus, the majority of genes within the modules negatively associated with *Salmonella* shedding counts had a higher mean expression in LS than PS at day 0 and were significantly up-regulated in both LS and PS at day 2 (Figure 4). However, the positively associated magenta module, in spite of showing a majority of genes expressed lower in LS than PS at day 0, did not show a trend of overall down-regulation at day 2 (figure not shown). In this case, we observed 3.1% (10 of 327) of the genes to be significantly down-regulated, 11.9% (39 of 327) significantly up-regulated, and the majority of the non-DE genes were more highly expressed at day 2.

### Differences in connectivity of *Salmonella* shedding associated genes between low and persistent shedders and between days

**Figure 5:**
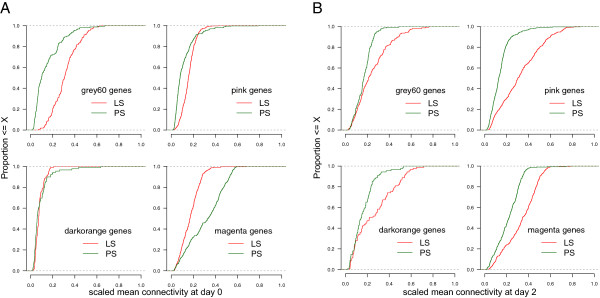
**Differential connectivity of genes within** ***Salmonella*** **shedding associated modules.** Connectivity measures of genes within *Salmonella* shedding associated modules in low and persistent shedders at day 0 **(A)**, and at day 2 **(B)**. The connectivity measures were normalised to the maximum connectivity within each network. The x-axis refers to the scaled mean connectivities of genes within a particular module in the LS and PS samples. The y-axis refers to the proportion of those genes whose scaled mean connectivities falls below a particular connectivity level.

Connectivity is a central concept in network statistics. In gene co-expression networks, connectivity measures how correlated a gene is with all other network genes. Here we calculated the scaled mean connectivities of all genes for four separate networks constructed using the following four subsets of the gene expression dataset: LS at day 0, PS at day 0, LS at day 2 and PS at day 2, and tested if the connectivities differed significantly between LS and PS (see Methods) when considering either all genes in the networks or only those genes belonging to the *Salmonella* shedding associated modules (which were obtained from the network constructed from all day 0 samples from both LS and PS). The mean, median and maximum connectivities were all higher for networks from PS samples than those from LS networks, at both days, when considering all genes in the networks. However, the opposite trend was seen when considering only the *Salmonella* responsive genes within each network. At day 0, the network connectivity measures were significantly higher in LS compared to PS for genes within the *pink* (p = 8.4e-27) and *grey60* (p = 2e-26) modules, not significantly different for the *darkorange* module genes and significantly lower in LS compared to PS for the *magenta* (p = 2e-26) module genes (Figure 5A). At day 2, the network connectivity measures were significantly higher in LS compared to PS for genes within all four *Salmonella* shedding associated modules (Figure 5B). Though significant differences in connectivity were also seen between LS and PS for some of the other modules not associated with *Salmonella* shedding both in the day 0 and day 2 networks, it was remarkable that the two immune function related modules, *pink* and *grey60*, showed significantly higher connectivities within the LS than PS even before *Salmonella* challenge.

### Prioritisation of candidate genes for *Salmonella* shedding

For prioritising candidate genes within the *Salmonella* shedding associated modules that could possibly determine better immune responses to *Salmonella* challenge, we restricted further analysis to the *pink* and *grey60* modules. These modules were found to be immune-related, had a majority of genes significantly up-regulated at day 2, were well preserved with the corresponding modules from the network constructed using day 2 samples, and also showed overall higher gene connectivity measures in LS compared to PS in networks from both days. A total of 213 genes in the *pink* module and 100 in the *grey60* module were up-regulated at day 2 p.i. Network properties of this refined set of candidate genes within these two modules are provided in Additional file 9. This candidate gene list includes genes previously reported to be involved in the regulation of host responses to *Salmonella* infection (*SLC11A1*, *TLR4*, *CD14*) or as having SNPs associated with *Salmonella* faecal shedding (*CCR1*), as well as those for which an association with *Salmonella* is novel (*SIGLEC5*, *IGSF6* and *TNFSF13B*).

**Figure 6:**
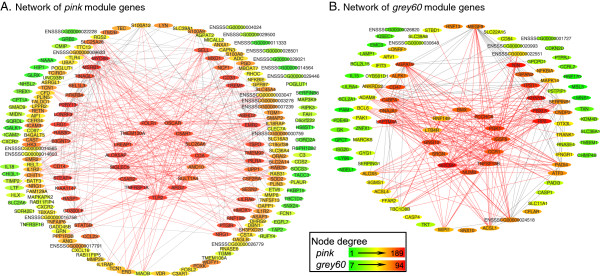
**Visualisation of the networks of selected modules associated with faecal** ***Salmonella*** **shedding.** A total of 213 genes in the *pink* module and 100 in the *grey60* module that were up-regulated at day 2 post inoculation are depicted in networks **A** and **B**. Nodes in the network are labelled by the corresponding pig/human ortholog gene symbols if available and pig Ensembl IDs if not. Each node is coloured based on degree (the number of connections it has to other genes in the network). To improve network visibility, the edges (connections) have been filtered to show only those with a correlation weight above 0.25 in the *pink* and 0.20 in the *grey60* module. The top candidate genes in each network as listed in Table 2 are displayed at the centre of the network with their edges coloured red.

We prioritised the genes within these refined sets of candidates based on module membership, gene significance and also connectivity in LS at day 0 since we speculated that the significantly higher gene connectivity in LS may have a biological significance related to *Salmonella* shedding. The top candidates in these modules with module membership above 0.9, gene significance below -0.4 and connectivity in LS at day 0 above 0.2, ordered by connectivity are presented in Table 2. These top candidates are also among the most well connected genes in the network visualisations using the refined set of candidate genes within the *pink* (Figure 6A) and *grey60* modules (Figure 6B).

**Table 2:** **Top candidate genes associated with** ***Salmonella*** **shedding**

***pink*** **module**							
**Ensembl ID**	**Gene symbol**	**GS**	**k**_**d0_LS**_	**k**_**d0_PS**_	**k**_**d2_LS**_	**k**_**d2_PS**_	**MM**
ENSSSCG00000022236	*FOLR1*	-0.56	0.29	0.06	0.41	0.18	0.93
ENSSSCG00000003259	*OSCAR*	-0.51	0.27	0.04	0.64	0.10	0.92
ENSSSCG00000029371	*C5AR1*	-0.49	0.27	0.07	0.49	0.16	0.92
ENSSSCG00000011361	*(SLC26A6)*	-0.57	0.26	0.06	0.74	0.14	0.92
ENSSSCG00000021259	*CDA*	-0.57	0.26	0.08	0.35	0.14	0.93
ENSSSCG00000011296	*ANO10*	-0.58	0.25	0.15	0.45	0.13	0.91
ENSSSCG00000021557	*(SULT1A4)*	-0.60	0.25	0.08	0.67	0.20	0.91
ENSSSCG00000002294	*ARG2*	-0.44	0.24	0.09	0.45	0.05	0.94
ENSSSCG00000009002	*TLR2*	-0.43	0.24	0.07	0.11	0.17	0.94
ENSSSCG00000000708	*TNFRSF1A*	-0.52	0.23	0.07	0.35	0.15	0.94
ENSSSCG00000003113	*C5AR2*	-0.61	0.23	0.12	0.70	0.09	0.93
ENSSSCG00000003236	*SIGLEC5*	-0.51	0.23	0.08	0.53	0.11	0.94
ENSSSCG00000009330	*ALOX5AP*	-0.47	0.23	0.07	0.57	0.06	0.94
ENSSSCG00000008701	*LREAP1*	-0.45	0.22	0.14	0.11	0.04	0.93
ENSSSCG00000021837	*TMEM120A*	-0.54	0.21	0.04	0.41	0.04	0.92
***grey60*** **module**							
**Ensembl ID**	**Gene symbol**	**GS**	**k**_**d0_LS**_	**k**_**d0_PS**_	**k**_**d2_LS**_	**k**_**d2_PS**_	**MM**
ENSSSCG00000012137	*BMX*	-0.45	0.48	0.10	0.46	0.09	0.94
ENSSSCG00000010952	*ZCCHC6*	-0.44	0.45	0.17	0.16	0.21	0.94
ENSSSCG00000012440	*PGK1*	-0.50	0.45	0.05	0.21	0.22	0.95
ENSSSCG00000027967	*IGSF6*	-0.48	0.45	0.06	0.22	0.15	0.95
ENSSSCG00000011322	*CCR1*	-0.49	0.38	0.07	0.50	0.21	0.95
ENSSSCG00000009542	*TNFSF13B*	-0.46	0.35	0.04	0.34	0.21	0.95
ENSSSCG00000022178	*(NUMB)*	-0.49	0.35	0.07	0.37	0.24	0.96
ENSSSCG00000006237	*SDCBP*	-0.44	0.32	0.17	0.13	0.27	0.96
ENSSSCG00000023374	*SRGN*	-0.44	0.31	0.19	0.41	0.18	0.95
ENSSSCG00000005462	*GNG10*	-0.45	0.29	0.07	0.40	0.24	0.95
ENSSSCG00000027911	*LTB4R*	-0.52	0.27	0.07	0.54	0.06	0.92
ENSSSCG00000008168	*RNF149*	-0.46	0.26	0.07	0.24	0.21	0.92

GS: gene significance, k _**d0_LS**_ and k _**d0_PS**_: gene connectivity in the day 0 network using low shedder samples and persistent shedder samples respectively and similarly k _**d2_LS**_ and k _**d2_PS**_ for the day 2 network, MM: module membership. Where the pig gene symbol is not available, the corresponding ortholog human gene symbol is provided.

Further, as an independent validation of our results, we compared the expression patterns of a subset of the candidate genes reported here with the corresponding expression patterns from a previous microarray based *Salmonella* challenge study involving a different set of LS and PS animals [20]. Probes on the microarray represented 19 (*FOLR1*, *SLC26A6*, *CDA*, *ANO10*, *ARG2*, *TLR2*, *TNFRSF1A*, *ALOX5AP*, *LREAP1*, *BMX*, *ZCCHC6*, *PGK1*, *CCR1*, *NUMB*, *SDCBP*, *SRGN*, *GNG10*, *LTB4R*, *RNF149*) of the top 27 candidate genes reported in Table 2. Except for *SLC26A6* and *NUMB*, all other genes showed expression patterns similar to those observed in our study: higher in LS than PS at day 0 and up-regulated in both LS and PS at day 2 p.i. In addition, the genes *SLC11A1*, *TLR4* and *CD14*, which are known to play important roles during *Salmonella* infection, showed similar expression patterns as described above. Figures comparing the expression patterns of these 22 genes between the two studies are provided in Additional file 10.

## Discussion

Significant gains have been made in our understanding of host–pathogen interactions during *Salmonella* infection [4]. Several studies, involving a variety of different species of farm and model animals, have investigated the host response to *Salmonella* infection and have successfully identified genes differentially expressed upon infection or gene variants and chromosomal loci associated with immune response traits during infection with *Salmonella*[7, 20, 27, 28]. A genetic basis for differences in resistance to *Salmonella* has also been shown with *SLC11A1* (*NRAMP1*), the seminal example of a gene with genetic variants dramatically affecting resistance to bacterial infection [29, 30]. While many studies have looked into the host response to *Salmonella* infection [4, 20, 28], relatively few have focused on identifying the genes whose variable expression among different individuals may be associated with differences in *Salmonella* clearance and resistance.

Initial characterisation of the pigs used in an earlier *Salmonella* challenge study [20] revealed a significant positive correlation between serum interferon-γ (IFN-γ) levels at day 2 p.i. and faecal *Salmonella* shedding levels at day 2 and day 7 p.i. The same study also demonstrated that the peak of both clinical symptoms (fever, diarrhea, decreased appetite) and *Salmonella* shedding occurs at day 2 p.i. and that substantial whole blood transcriptome changes occur at day 2 p.i. compared to day 0 in pigs belonging to both LS and PS groups. Therefore, here we chose to profile whole blood transcriptomes at the same time-points, day 0 and day 2, but belonging to a different set of pigs, using RNA-Seq instead of microarrays, for a different purpose: to identify genes whose expression prior to inoculation is correlated with *Salmonella* shedding levels observed p.i. The genes identified may serve as blood-based candidate biomarkers that could potentially be used to develop quick screening tests for determining the host’s resistance/susceptibility to *Salmonella* infection and predicting their shedding characteristics early into or even before infection.

The extent of DE and degree of change in expression between day 0 and day 2 were, in general, higher in PS than LS. This finding, as previously speculated [20], may indicate that LS animals respond faster and more effectively against infection than PS so that by day 2, while the LS are returning back to normal, the PS are still actively fighting the infection. With this in mind we believe that the comparison between LS and PS at day 2 likely identifies DE due to differing levels of infection. A comparison at day 0, on the other hand, stands to better highlight genes responsible for differences in the efficacy of the initial response to the bacteria, assuming that some of these genes exhibit expression differences prior to infection. However, the DE analysis between LS and PS at day 0 did not yield any significant DE genes here as well as in an earlier *Salmonella* challenge study [20]. This failure to find DE genes could be due to a combination of the subtlety of the expression differences, the relatively small sample size, and the strict multiple testing corrections. Hence we used an alternative approach, WGCNA, to find genes associated with *Salmonella* shedding.

Remarkably, the genes whose pre-inoculation expression profiles were found associated with post-inoculation *Salmonella* shedding levels included major genes already reported in literature as DE during *Salmonella* infection and involved with host resistance against *Salmonella* such as *SLC11A1*, *TLR4*, *CD14* and *CCR1*[4, 20]. Moreover, the majority of genes within the modules significantly associated with *Salmonella* shedding, following further refinement based on up-regulation and DE at day 2 (Additional file 9), were found to have an established or possible role in innate defense against bacterial/*Salmonella* infections. These include mainly the early innate immune response genes responsible for cytokine-cytokine receptor interactions (*CXCL16*, *CCR1*, *CCR3*, *CNTF*, *CSF2RA*, *IFNGR1*, *TNFSF9*, *TNFSF12*, *TNFSF13*, *TNFSF13B*, *TNFRSF1A*, *TNFRSF1B*, *IL1A*, *IL15*, *IL18*, *IL1RAP*, *IL1R2*, *IL18RAP*), genes involved in toll-like receptor pathway (*TLR4*, *TLR2*, *MYD88*, *CD14*, *LY96*), NF-Kappa B signalling pathway (*NFKBIA*, *TNFRSF1A*, *TNFSF13B*), NOD-like receptor signalling pathway (*CASP1*, *PSTPIP1*, *IL18*, *NFKBIA*) or otherwise linked to response to bacterial infections (*SLC11A1*, *SERPINB1*, *S100A8*, *S100A9*, *S100A12*, *ARG2*, *CEBPB*). Prioritisation of these genes based on gene significance, module membership and gene connectivity in LS at day 0 (Table 2) highlights some genes which have not been previously associated with *Salmonella* infection. For example, little is known about the role of *SIGLEC5*, a member of the Siglec family of sialic acid-binding lectins in host response to bacterial infection. However, it has been reported in humans that the absence of a functional *SIGLEC14* (with which human *SIGLEC5* shows extensive sequence similarity) results in attenuated cytokine response to some Gram-negative bacteria in null individuals [31].

The mean expression levels of the *Salmonella* shedding associated genes at day 0 were generally higher in LS than PS and mostly up-regulated in both at day 2 compared to day 0. In most instances, the expression was higher in PS than LS at day 2. We showed, at least for the top candidate genes reported here, that the pattern of expression is consistent with that from a previous microarray based *Salmonella* challenge study involving a different set of LS and PS animals. Examining the connectivities of genes within the *Salmonella* shedding associated modules in LS and PS, it became apparent that the genes in general showed higher connectivity in LS than PS, indicative of higher correlation/connection strengths with other network genes. The differences in connectivity measures for a set of genes between different conditions may signify differences in the co-ordination or strength of transcriptional regulation of that set of genes. Highly connected genes (hub genes) have been shown to play central roles in the biological processes that are represented by the module [32], and strong positive correlations have been reported between gene connectivity within the whole network and gene essentiality [33]. Here, the significantly higher connectivity despite the lack of significant DE between LS and PS may be considered analogous to the results in a study on breast cancers of different histological grades [34]. The authors of that study concluded that the differential connectivity patterns were not due to primary alterations of hub gene expression, but rather due to more subtle changes in expression of numerous genes interacting with those hubs. Further, they reported that complex epistatic interactions that underlie cellular functions might also be responsible for differences in network connectivity patterns as a function of a phenotypic trait. A study on aging in mice [35] reported a decreasing correlation of gene expression within genetic modules and attributed this to changes in expression of certain transcription factors (TF) as well as deterioration of chromatin structure with age. It is possible that genetic differences at mutiple levels as discussed above may contribute to the differences in strengths of coexpression and connectivity between LS and PS. Exploring these contributions may be a direction for future research.

One of the limitations of our study is the absence of samples from time points post-inoculation but before day 2 p.i., which are crucial to capture the early immune response during which the LS pigs have effectively managed the *Salmonella* challenge. Secondly, this study would benefit from a larger sample size, which would provide more power to detect the subtle changes in expression expected between LS and PS animals. Further experiments are required to rank the relative functional importance of our suggested candidate genes as contributors to distinct responses to *Salmonella* challenge with respect to faecal *Salmonella* shedding. However, the use of multiple criteria and strict cut-offs to refine the set of candidate genes, the agreement with existing literature regarding the immune related functions of many candidate genes and the concordance of the expression patterns of top candidate genes reported here with the corresponding expression patterns from an independent dataset, all lend further support to our predictions.

## Conclusions

Our analysis integrates gene co-expression network analysis, gene-trait correlations and differential expression to provide new candidate regulators of *Salmonella* shedding in pigs with implications for future use as biomarkers for selection of animals with reduced susceptibility to, or carriage of, *Salmonella* or for predicting response to infection. The comparatively higher expression (also confirmed in an independent dataset) and the significantly higher connectivity of genes within the *Salmonella* shedding associated modules in LS compared to PS even before *Salmonella* challenge may be factors that contribute to the decreased faecal *Salmonella* shedding observed in LS following challenge.

## Methods

### Sample collection, *Salmonella* shedding status determination and selection of animals for RNA-Seq analysis

Samples used in this study were selected from *Salmonella* negative piglets (crossbred or Yorkshire sows bred to boars from different breeds) belonging to two populations of 40 and 77 individuals and treated as described in an earlier study from our group [36]. In brief, the pigs were raised in climate-controlled, fully enclosed isolation facilities at the USDA-ARS-National Animal Disease Center (NADC) in Ames, IA under identical management conditions. At 7 weeks of age, these *Salmonella* negative pigs received intranasal inoculation of 10^9^ colony-forming units (cfu) of nalidixic acid resistant *Salmonella enterica* serovar Typhimurium, ST χ4232. Approximately 2.3 ml of whole blood was collected from the jugular vein into PAXgene Blood RNA tubes (processed according to manufacturer’s instructions) from each individual just prior to *Salmonella* inoculation (day 0) and at days 2, 7, 14, and 20 p.i. On the same days, faecal samples were also collected and the amount of *Salmonella* bacteria shed in faeces was quantified by direct counting using bacteriological methods as described by Uthe et al. [12]. All procedures involving animals were approved by the USDA-ARS-NADC Animal Care and Use Committee.

*Salmonella* shedding status was determined based on the total amount of *Salmonella* shed in faeces calculated using the cumulative area under the plotted log curve (AULC) of the logarithmically normalised faecal counts obtained between day 0 to day 20 post-inoculation for each individual as described in earlier publications [12, 20]. Based on the AULC, 16 pigs were selected for RNA-Seq analysis, eight of which were identified as LS and eight as PS.

### RNA preparation, library construction and Illumina sequencing

Blood samples collected at day 0 and day 2 p.i. from 16 selected pigs identified as LS (n = 8) and PS (n = 8) were used for total RNA extraction. Total RNA was prepared from 4.5–9.0 ml of solution from the PAXgene Blood RNA tubes and extracted using the PAXgene Blood miRNA Kit (Qiagen) according to the manufacturer’s instructions. The DNA was removed by in-solution DNase I digestion and RNeasy MinElute Cleanup Kit as recommended by Qiagen. A PCR assay without reverse transcription was used to confirm that the RNA samples were DNA-free. The quantity and quality of the RNA were determined using the Agilent 2100 Bioanalyzer (Agilent Technologies, Santa Clara, CA) and Nanodrop 2000 (Thermo Scientific, Wilmington, DE).

A globin reduction protocol (GR) developed in-house [25] using porcine specific oligonucleotides was followed. A 10X GR oligonucleotide mix was prepared by combining the four oligos (two each for HBA and HBB; see Additional file 11 for details) to the final concentrations of 7.5 uM, 7.5 uM, 30 uM and 30 uM, respectively. Next, 2 uL of the 10X GR oligonucleotide mix was then added to 3 ug (7 uL) of total RNA and 1 uL of 10X oligonucleotide hybridisation buffer (100 mM Tris–HCl, pH 7.6; 200 mM KCl) to constitute the 10 uL of hybridisation mix. This mix was incubated in a thermal cycler at 70°C for 5 minutes and then cooled to 4°C. The RNA-DNA hybrids were digested with 2 U RNase H (Ambion) in the reaction buffer (100 mM Tris–HCl, pH 7.6, 20 mM MgCl_2_, 0.1 mM DTT, SUPERase-in) at 37°C for 10 minutes and cooled to 4°C. The reaction was stopped by addition of 0.5 M EDTA. The globin depleted RNA was immediately purified with the RNeasy MinElute Cleanup Kit (Qiagen, Toronto, Canada, Cat. No.: 74204) according to manufacturer’s instructions. RNA quality of the globin depleted samples was assessed using an Agilent Bioanalyzer 2100 (Agilent Technologies, Inc., Santa Clara, USA).

The mRNA library was constructed using the TruSeq RNA Sample Preparation Kit v2 (Illumina, Inc., San Diego, USA) according to manufacturer’s instructions. One *u*g of total RNA was purified using poly-T oligo-attached magnetic beads. The poly (A) RNA was primed and fragmented. The first strand and second strand cDNA were synthesised according to the Illumina sample preparation guide. The adapters with different indices were ligated to the cDNA fragments and the library was subsequently PCR-amplified. The qualities of the mRNA libraries were confirmed using the Agilent Bioanalyzer 2100 (Agilent Technologies, Inc.). Quantification was performed using the StepOne Real-Time PCR System (Applied Biosystems, Carlsbad, USA). The individual libraries were adjusted to 2 nM concentrations and pooled before denaturation and dilution according to Illumina’s instructions. The diluted libraries (8 – 10 pM) were loaded on a cBot (Illumina) for cluster generation using the TruSeq SR Cluster Kit v3 (Illumina). Sequencing was performed on the HiScanSQ system (Illumina) using the TruSeq SBS Kit v3 (50 cycles, single-read sequencing, from Illumina). Real-time analysis and base calling were performed using the HiSeq Control Software, version 1.4.8 (Illumina).

### Identification and quantification of mRNAs

Sequence reads with base quality scores were produced by the Illumina sequencer. RNA-Seq reads flagged as low quality by the chastity filter in CASAVA 1.8 were removed. In addition, we removed reads with an average read quality score below 15 and reads in which over 5 of the last 10 bases had a PHRED quality score below 2. Pig reads were aligned to pig reference genome sequence assembly (Sscrofa10.2) [37] using Tophat 1.4.0 [38] with default parameters which included: enable use of GTF file, set minimum anchor length of 8, accept zero mismatches in the anchor region, allow intron length between 50 and 500,000, and allow up to 20 alignments to the reference for a given read. For annotation of genes, we used the GTF file for Sscrofa10.2 from Ensembl version 67 [39]. The number of reads uniquely mapped to each gene was determined using Htseq-count (v0.5.3.p3) [40]. Reads that were assigned to more than one gene were not counted by Htseq-count. For further processing of the read counts, we used the Bioconductor (version 2.18.0) [41] package edgeR (version 3.0.8) [42] within the R (version 2.15.2) statistical programming language [43]. The read counts per gene were normalised to counts per million (CPM). Genes expressed at very low levels were removed by keeping only those genes that achieve CPM above one in at least eight samples. Trimmed mean of M-values (TMM) normalisation was applied to this dataset to account for compositional differences between the libraries. Gene expression was also quantified as reads per kilobase of transcripts per million mapped reads (RPKM), calculated by dividing CPM by the respective gene lengths expressed in kilobase pairs. The gene lengths were taken as the length of the longest transcript for the respective genes obtained from the GTF file for Sscrofa10.2. CPM values were used for the DE analysis whereas RPKM values were used for the co-expression analysis. Clustering of samples by gene expression data (transformed to log2 CPM) was evaluated using multi-dimensional scaling in two dimensions using the *plotMDS* function in the limma (version 3.14.4) [44] package of Bioconductor. This function considers the Euclidian distance between each pair of samples for the top 500 genes with the largest standard deviations between samples i.e. the leading log2-fold-changes.

### Differential expression and co-expression network analyses

DE analysis was performed using the Bioconductor package edgeR and gene co-expression network analysis was performed using the R package WGCNA (version 1.26) [21]. The co-expression analysis starts by constructing a matrix of pairwise correlations between all pairs of genes across all selected samples. Next the matrix is raised to a soft-thresholding power (β = 8 in this study) to obtain an adjacency matrix. In order to identify modules of co-expressed genes, we construct the topological overlap-based dissimilarity [45, 46], which is then used as input to average linkage hierarchical clustering. This step results in a clustering tree (dendrogram) whose branches are identified for cutting depending on their shape using the dynamic tree-cutting algorithm [47] which has several advantages over the constant height cut-off method including the ability to identify nested clusters. Modules whose eigengenes are highly correlated are merged. The above steps were performed using the automatic network construction and module detection function (*blockwiseModules* in WGCNA), with the following major parameters: *maxBlockSize* of 11000, *minModuleSize* of 30, *reassignThreshold* of 0 and *mergeCutHeight* of 0.15. The robustness of the network modules was evaluated using a bootstrap approach implemented with functions available in WGCNA. Using the same parameters as described above, we performed 50 full module constructions and module detection runs on resampled datasets where in each run, 16 samples were selected randomly (with replacement) from the 16 day 0 samples. The module assignments in these runs were compared with the modules in the original network. Next, the modules were tested for their associations with the trait by correlating module eigengenes with trait measurements, AULC of faecal *Salmonella* shedding counts in this study. We used the *softconnectivity* function within the WGCNA package to calculate the connectivities of all genes in a network, which were then scaled to the maximum connectivity within that network. Differences between the scaled mean connectivities of selected genes of interest between different networks (constructed using subsets of the gene expression dataset) were tested using the Wilcoxon Rank Sum test. Detailed tutorials with examples for the use of the WGCNA method can be found at http://labs.genetics.ucla.edu/horvath/CoexpressionNetwork/Rpackages/WGCNA/Tutorials.

The gene co-expression networks for selected modules of genes were visualised using Cytoscape (version 3.1.0) [48–50]. The following Bioconductor packages were used for gene annotations: biomaRt version (2.14.0) [51], AnnotationDbi (version 1.20.7) and org.Hs.eg.db (version 2.8.0). Gene ontology (GO) [52] term enrichment tests were performed for individual gene co-expression modules compared to a background set of all genes expressed in blood using the Bioconductor packages GOstats (version 2.26.0) and GSEABase (version 1.22.0). The human orthologs of the corresponding porcine genes were used in the GO enrichment tests to take advantage of the more complete GO annotation available for human genes. Functional classification of porcine blood expressed genes based on GO terms from GO Slim (a subset of GO terms that gives a broad overview of the GO ontology) was performed using the PANTHER gene list analysis tool (version 8.1, June 2013) [53, 54].

### Availability of supporting data

The RNA-Seq data supporting the results of this article are available in the ArrayExpress database [55] (http://www.ebi.ac.uk/arrayexpress) under accession number E-MTAB-2234.

## Electronic supplementary material

Additional file 1: **Sequencing depth and mapping statistics.** PDF file contains bar plot indicating counts (in millions) of reads mapped out of the total reads sequenced per sample. (PDF 54 KB)

Additional file 2: **Proportion of globin reads among total mapped reads post globin depletion treatment.** PDF file contains bar plot indicating percentages of globin reads among total mapped reads per sample post globin depletion treatment. (PDF 31 KB)

Additional file 3: **Multi-dimensional scaling plot of gene expression dataset.** PDF file contains multi-dimensional scaling plot showing samples clearly separated by days but not by shedding statuses on either day. (PDF 28 KB)

Additional file 4: **Gene ontology based functional classification of genes expressed in porcine whole blood.** PDF file contains bar plot indicating percentages of expressed genes annotated to PANTHER GO Slim terms. (PDF 34 KB)

Additional file 5: **Module stability analysis from bootstrapped networks.** PDF file depicts the gene dendrogram for the original co-expression network constructed from day 0 samples and the module labels from resampled data. (PDF 3 MB)

Additional file 6: **Genes within the** ***Salmonella*** **shedding associated modules.** Excel file contains the Ensembl gene IDs of all genes within the four modules associated with faecal *Salmonella* shedding counts. (XLSX 71 KB)

Additional file 7: **Gene ontology terms enriched in** ***Salmonella*** **shedding associated modules.** Excel file contains gene ontology biological process terms enriched in modules associated with faecal *Salmonella* shedding counts. (XLSX 42 KB)

Additional file 8: **Heat maps of differentially expressed genes upon** ***Salmonella*** **challenge.** PDF file contains heat maps of differentially expressed genes for the day 0 versus day 2 comparison using all samples from low and persistent shedders. (PDF 93 KB)

Additional file 9: **Network properties of the refined set of** ***Salmonella*** **shedding associated genes.** Excel file contains the gene significance, module membership and network connectivity measures for the refined set of genes identified as both associated with *Salmonella* shedding before inoculation and differentially expressed at day 2 post inoculation. (XLSX 29 KB)

Additional file 10: **Comparison of the expression patterns of candidate genes associated with** ***Salmonella*** **shedding in an independent microarray dataset.** PDF file shows the concordance of the expression patterns of a subset of the candidate genes associated with *Salmonella* shedding reported in this study with the corresponding expression patterns from an earlier microarray based *Salmonella* challenge study using a different set of animals. (PDF 36 KB)

Additional file 11: **Porcine specific globin oligonucleotides used in the globin reduction protocol.** Excel file provides the sequences of the oligonucleotides used for the *HBA* and *HBB* globin reduction protocol. (XLSX 35 KB)
