# Supplementary figures and images for: Gene co-expression network analysis identifies porcine genes associated with variation in Salmonella shedding
Source: BMC Genomics. 2014 Jun 9;15(1):452. doi: 10.1186/1471-2164-15-452 (PMC4070558; doi:10.1186/1471-2164-15-452)

# Sequencing depths and mapping statistics

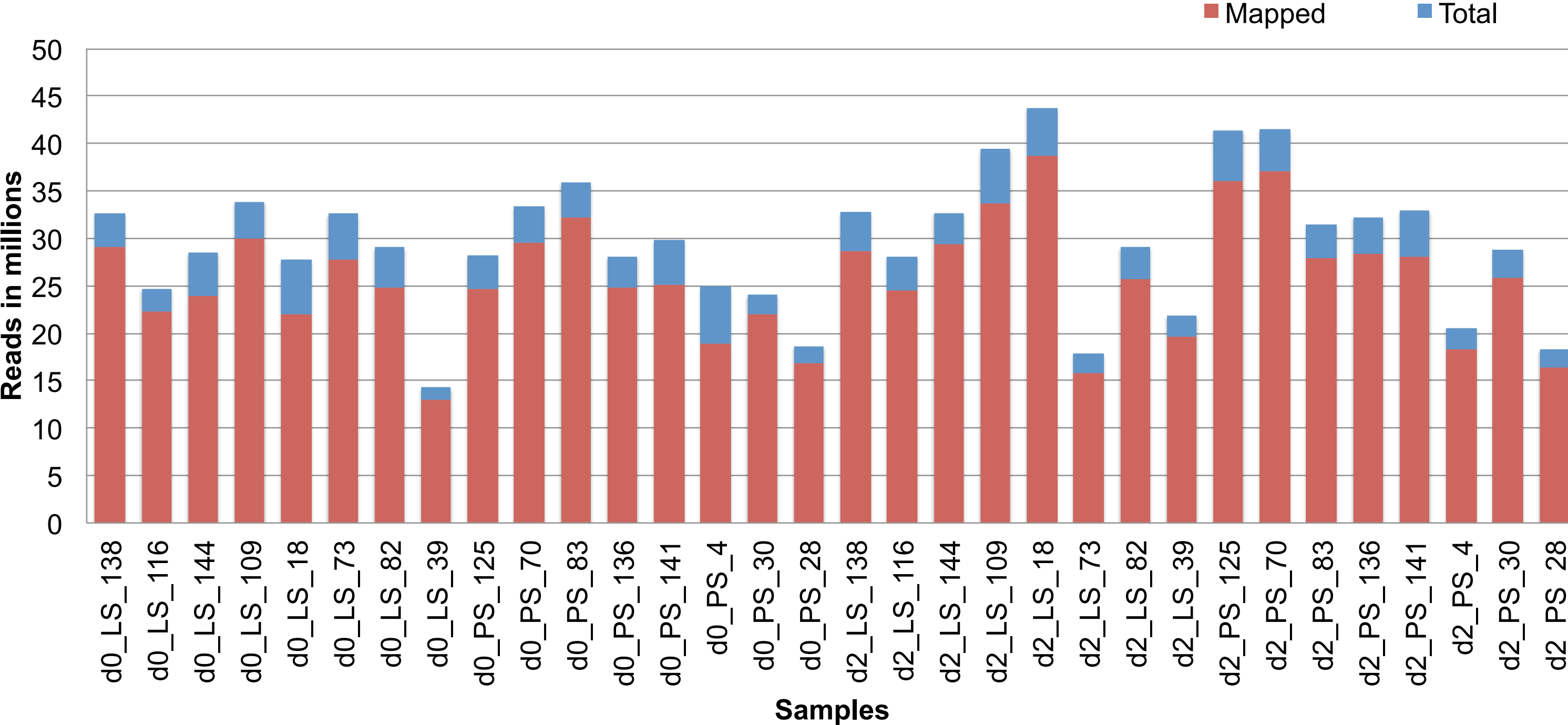

Supplement: Supplementary file 1 — Additional file 1: Sequencing depth and mapping statistics. PDF file contains bar plot indicating counts (in millions) of reads mapped out of the total reads sequenced per sample. (PDF 54 KB) [file 12864_2014_6126_MOESM1_ESM.pdf]

# Proportion of globin reads among total mapped reads post globin depletion treatment

HBA HBB

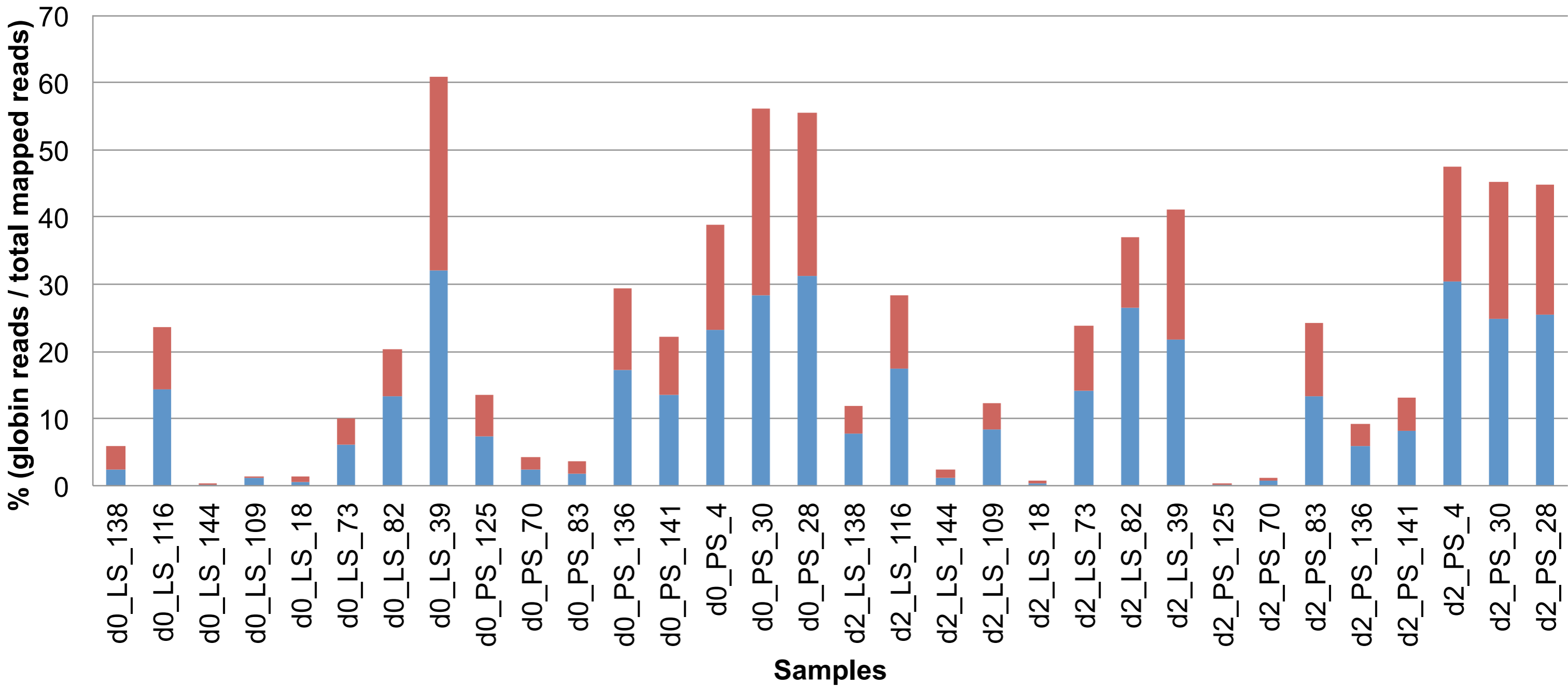

Supplement: Supplementary file 2 — Additional file 2: Proportion of globin reads among total mapped reads post globin depletion treatment. PDF file contains bar plot indicating percentages of globin reads among total mapped reads per sample post globin depletion treatment. (PDF 31 KB) [file 12864_2014_6126_MOESM2_ESM.pdf]

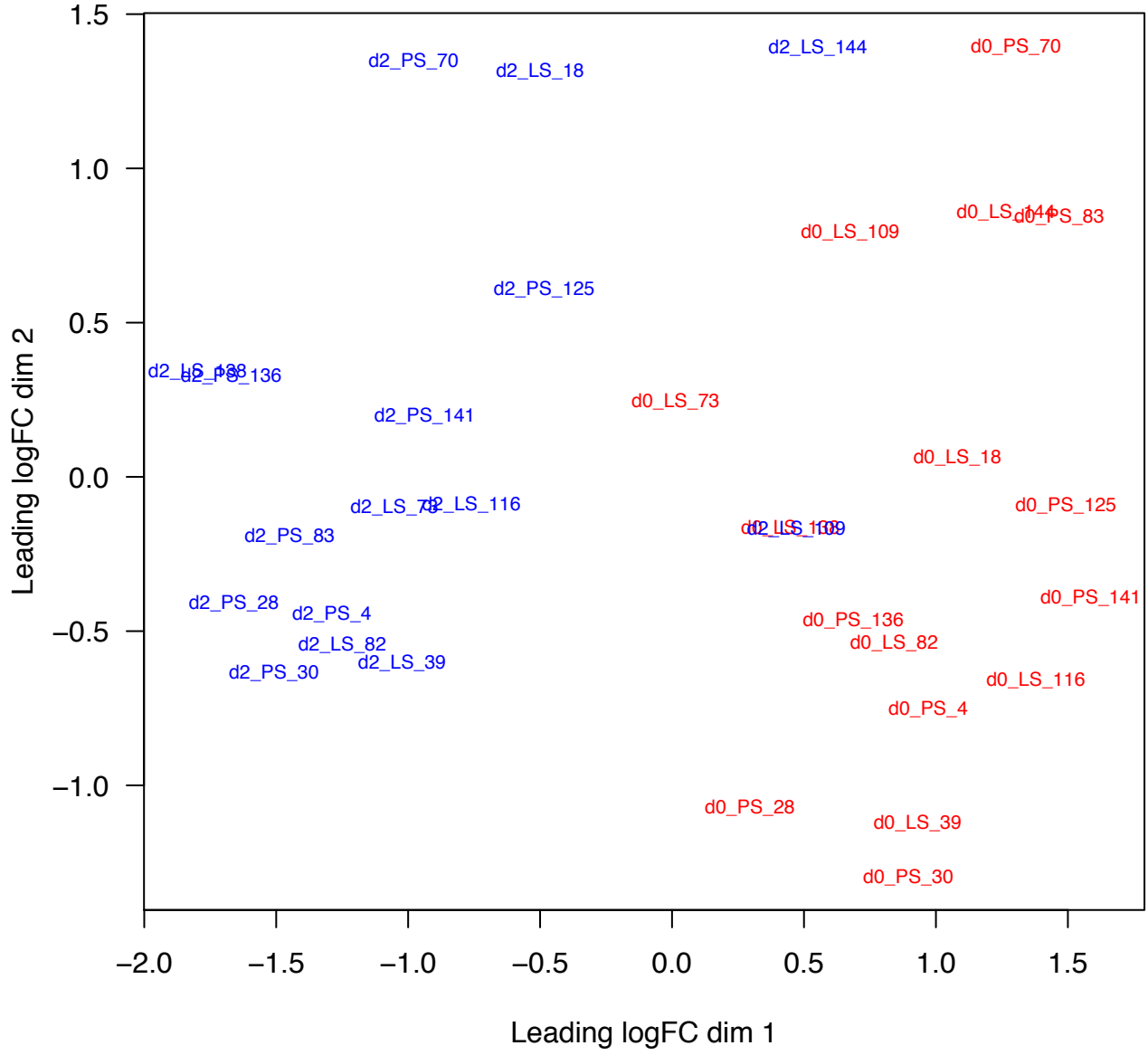

Supplement: Supplementary file 3 — Additional file 3: Multi-dimensional scaling plot of gene expression dataset. PDF file contains multi-dimensional scaling plot showing samples clearly separated by days but not by shedding statuses on either day. (PDF 28 KB) [file 12864_2014_6126_MOESM3_ESM.pdf]

## Gene Ontology Biological Processes

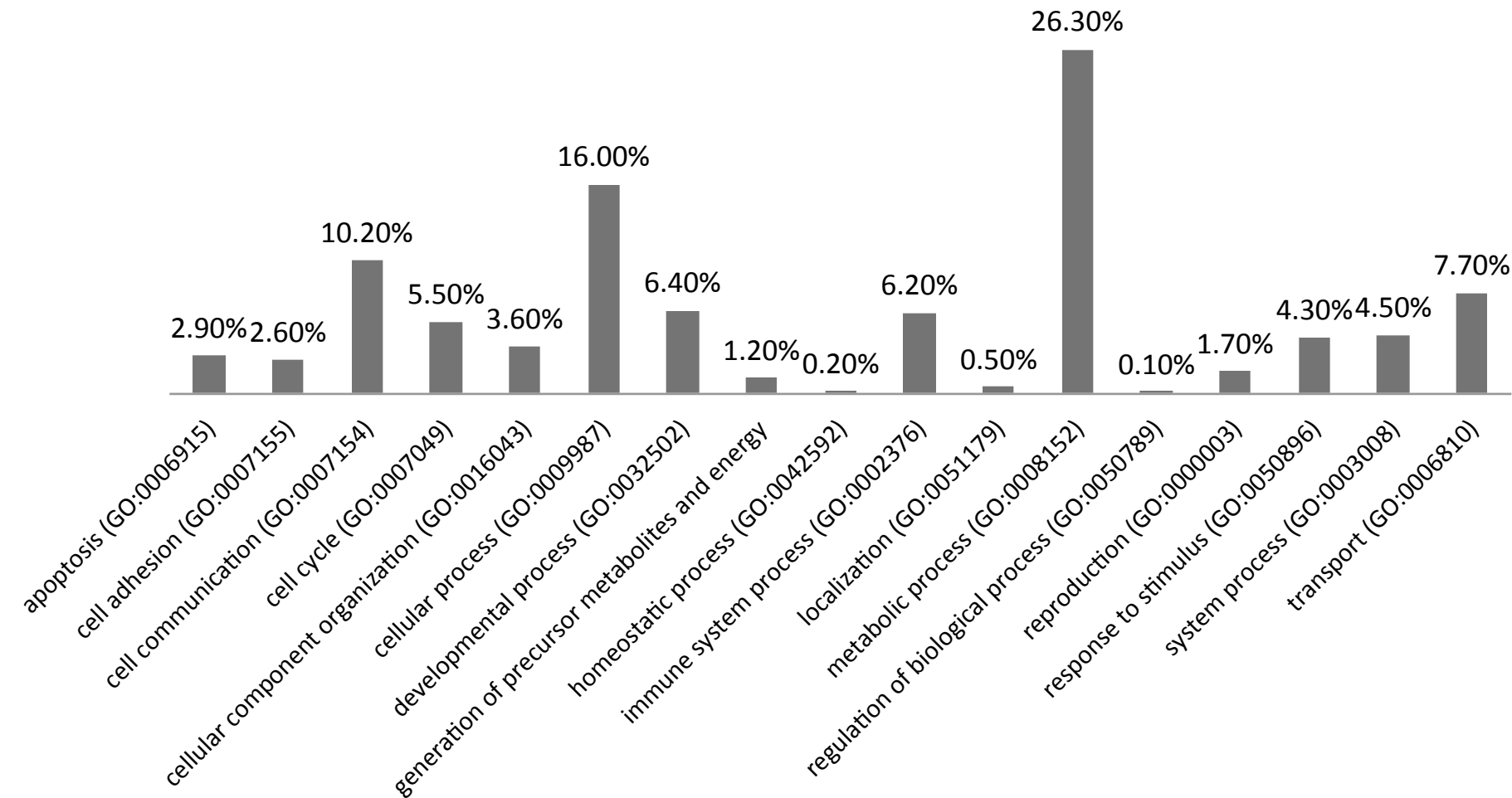

Supplement: Supplementary file 4 — Additional file 4: Gene ontology based functional classification of genes expressed in porcine whole blood. PDF file contains bar plot indicating percentages of expressed genes annotated to PANTHER GO Slim terms. (PDF 34 KB) [file 12864_2014_6126_MOESM4_ESM.pdf]

# Gene dendrogram and module labels from resampled datasets

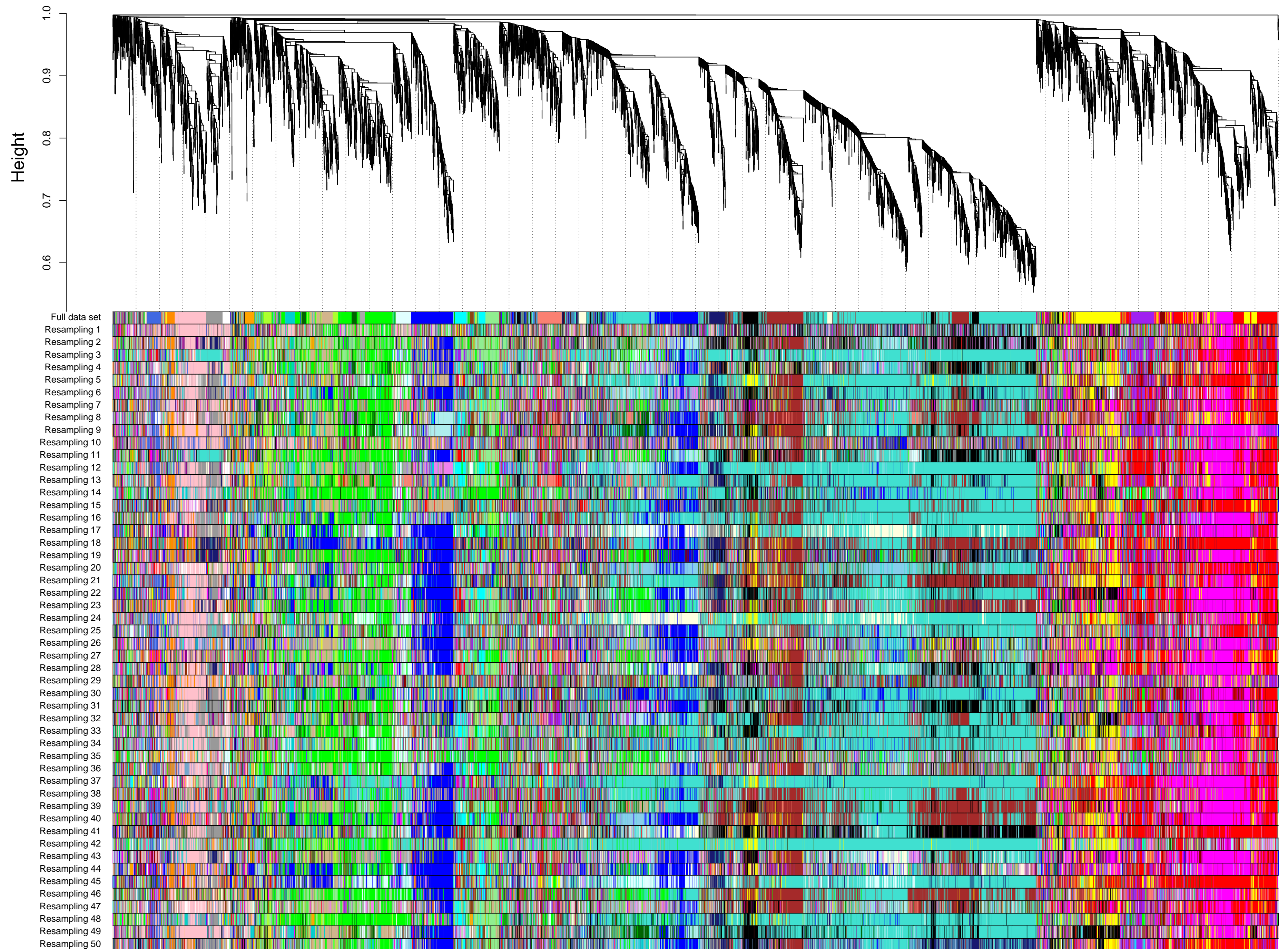

Supplement: Supplementary file 5 — Additional file 5: Module stability analysis from bootstrapped networks. PDF file depicts the gene dendrogram for the original co-expression network constructed from day 0 samples and the module labels from resampled data. (PDF 3 MB) [file 12864_2014_6126_MOESM5_ESM.pdf]
