# Supplementary material for: Gene co-expression network analysis identifies porcine genes associated with variation in Salmonella shedding
Source: BMC Genomics. 2014 Jun 9;15(1):452. doi: 10.1186/1471-2164-15-452 (PMC4070558; doi:10.1186/1471-2164-15-452)
Supplement: Supplementary file 8 — Additional file 8: Heat maps of differentially expressed genes upon Salmonella challenge. PDF file contains heat maps of differentially expressed genes for the day 0 versus day 2 comparison using all samples from low and persistent shedders. (PDF 93 KB) [file 12864_2014_6126_MOESM8_ESM.pdf]

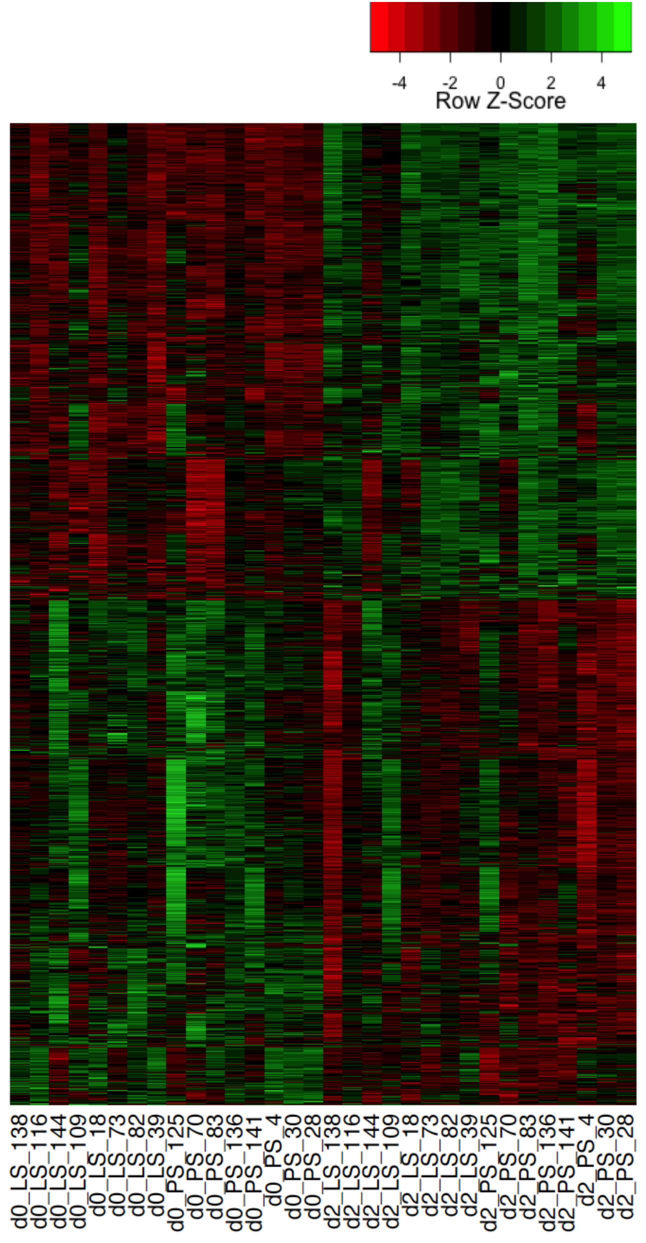

-4
-2
0
2
4
Row Z-Score
d0_LS_138
d0_LS_116
d0_LS_144
d0_LS_109
d0_LS_18
d0_LS_73
d0_LS_82
d0_LS_39
d0_PS_125
d0_PS_70
d0_PS_83
d0_PS_136
d0_PS_141
d0_PS_4
d0_PS_30
d0_PS_28
d2_LS_138
d2_LS_116
d2_LS_144
d2_LS_109
d2_LS_18
d2_LS_73
d2_LS_82
d2_LS_39
d2_PS_125
d2_PS_70
d2_PS_83
d2_PS_136
d2_PS_141
d2_PS_4
d2_PS_30
d2_PS_28
