# Supplementary material for: Gene co-expression network analysis identifies porcine genes associated with variation in Salmonella shedding
Source: BMC Genomics. 2014 Jun 9;15(1):452. doi: 10.1186/1471-2164-15-452 (PMC4070558; doi:10.1186/1471-2164-15-452)
Supplement: Supplementary file 10 — Additional file 10: Comparison of the expression patterns of candidate genes associated with Salmonella shedding in an independent microarray dataset. PDF file shows the concordance of the expression patterns of a subset of the candidate genes associated with Salmonella shedding reported in this study with the corresponding expression patterns from an earlier microarray based Salmonella challenge study using a different set of animals. (PDF 36 KB) [file 12864_2014_6126_MOESM10_ESM.pdf]

FOLR1

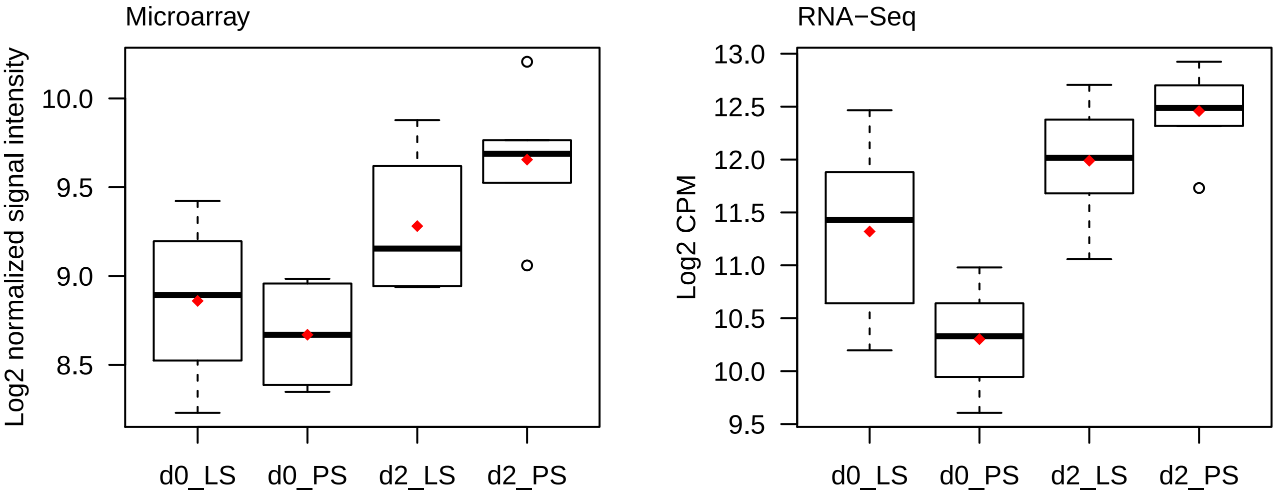

SLC26A6

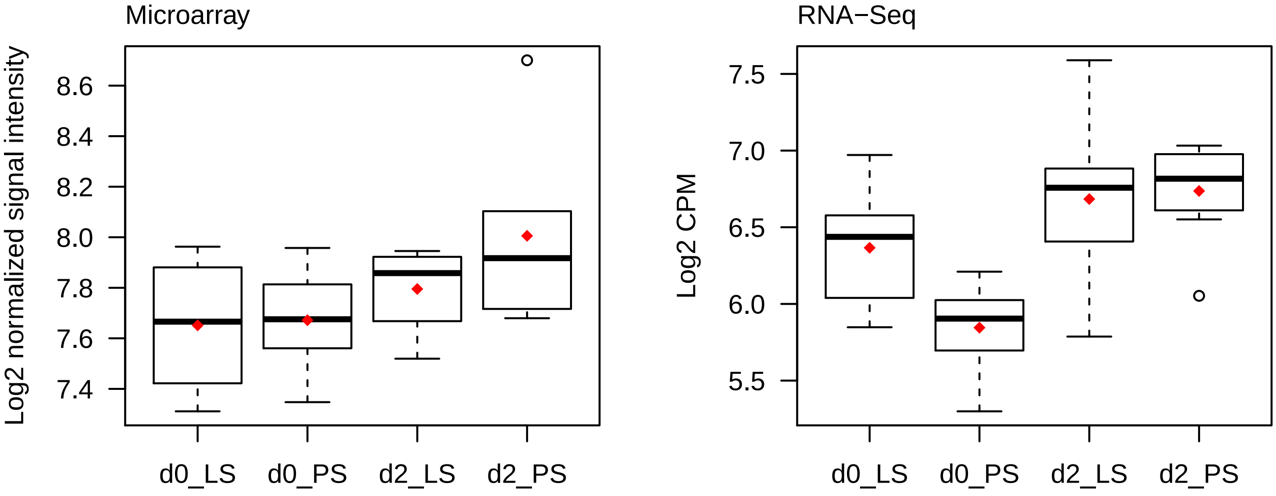

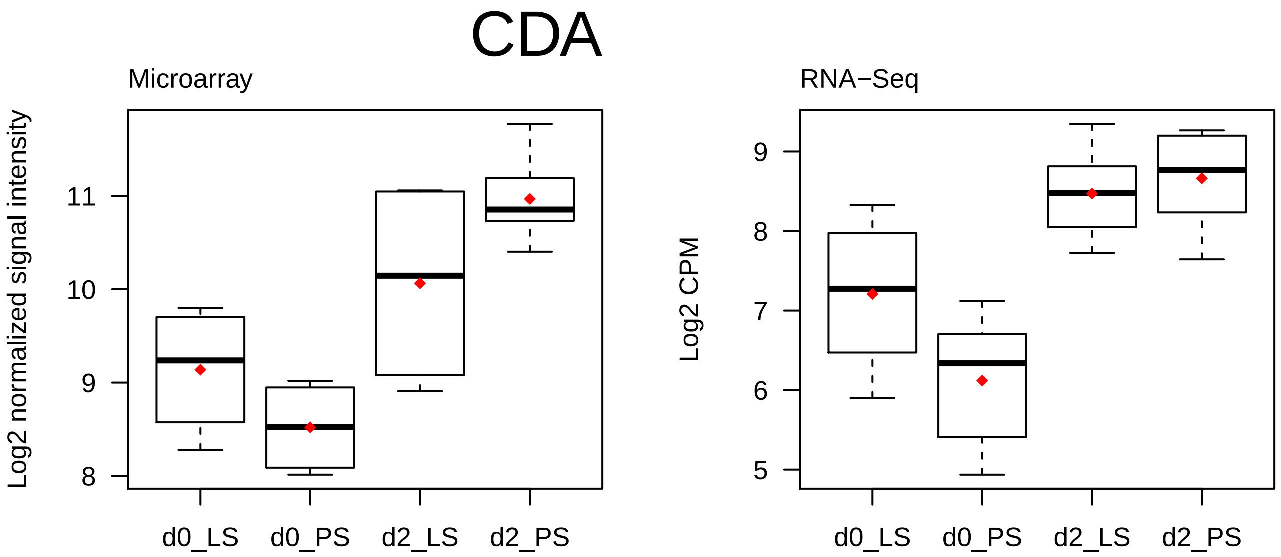
CDA
Microarray
Log2 normalized signal intensity
8
9
10
11
d0_LS
d0_PS
d2_LS
d2_PS
RNA−Seq
Log2 CPM
5
6
7
8
9
d0_LS
d0_PS
d2_LS
d2_PS

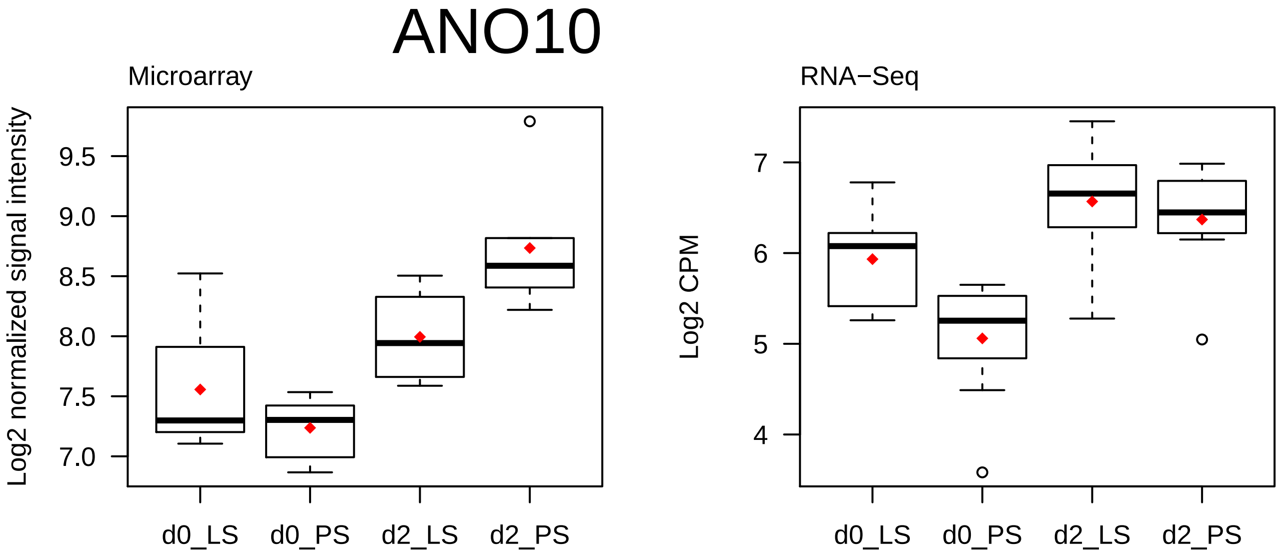
ANO10
Microarray
Log2 normalized signal intensity
7.0
7.5
8.0
8.5
9.0
9.5
d0_LS
d0_PS
d2_LS
d2_PS
RNA−Seq
Log2 CPM
4
5
6
7
d0_LS
d0_PS
d2_LS
d2_PS

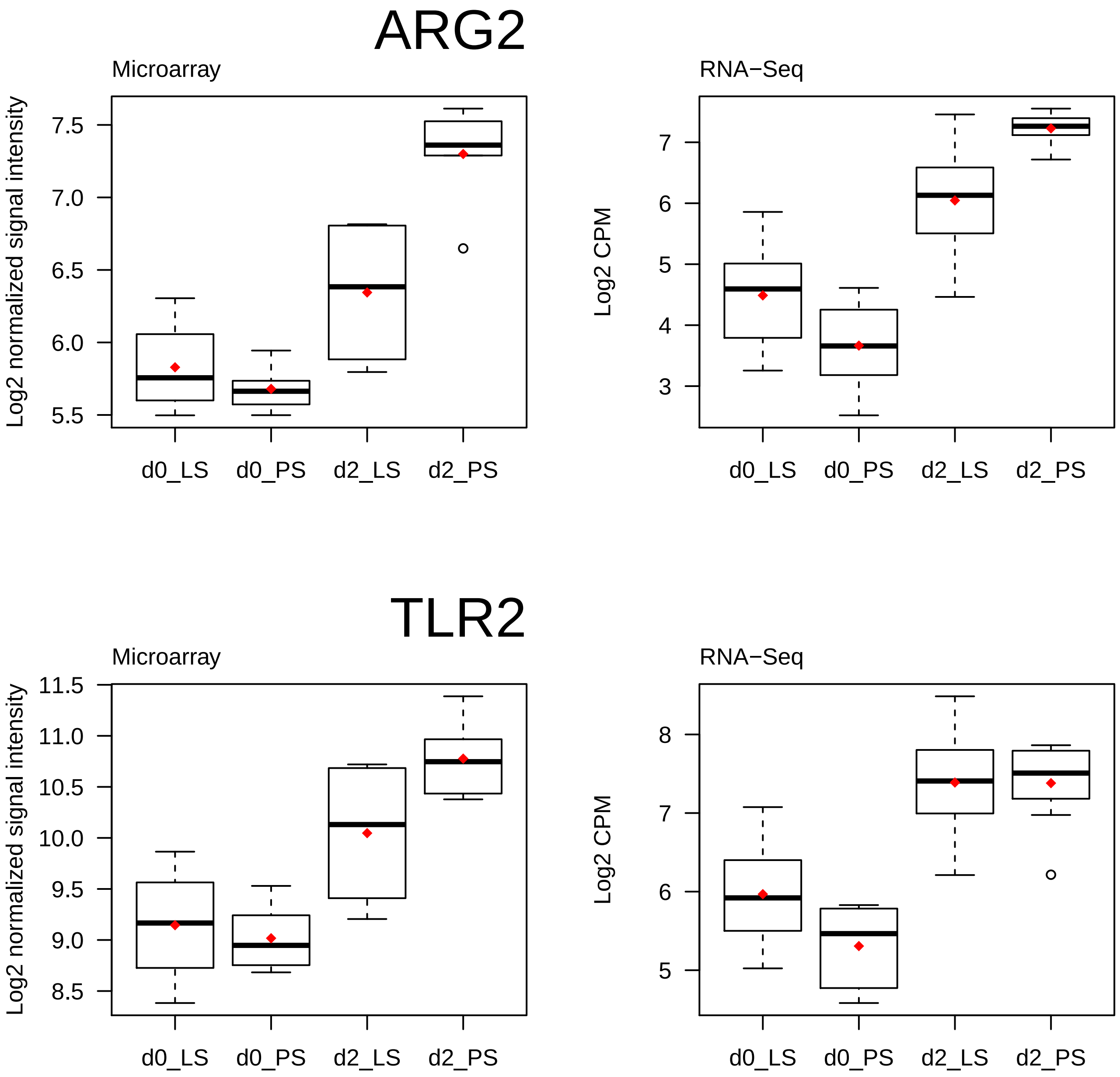
ARG2
Microarray
RNA−Seq
Log2 normalized signal intensity
Log2 CPM
5.5
6.0
6.5
7.0
7.5
3
4
5
6
7
d0_LS
d0_PS
d2_LS
d2_PS
TLR2
Microarray
RNA−Seq
Log2 normalized signal intensity
Log2 CPM
8.5
9.0
9.5
10.0
10.5
11.0
11.5
5
6
7
8
d0_LS
d0_PS
d2_LS
d2_PS

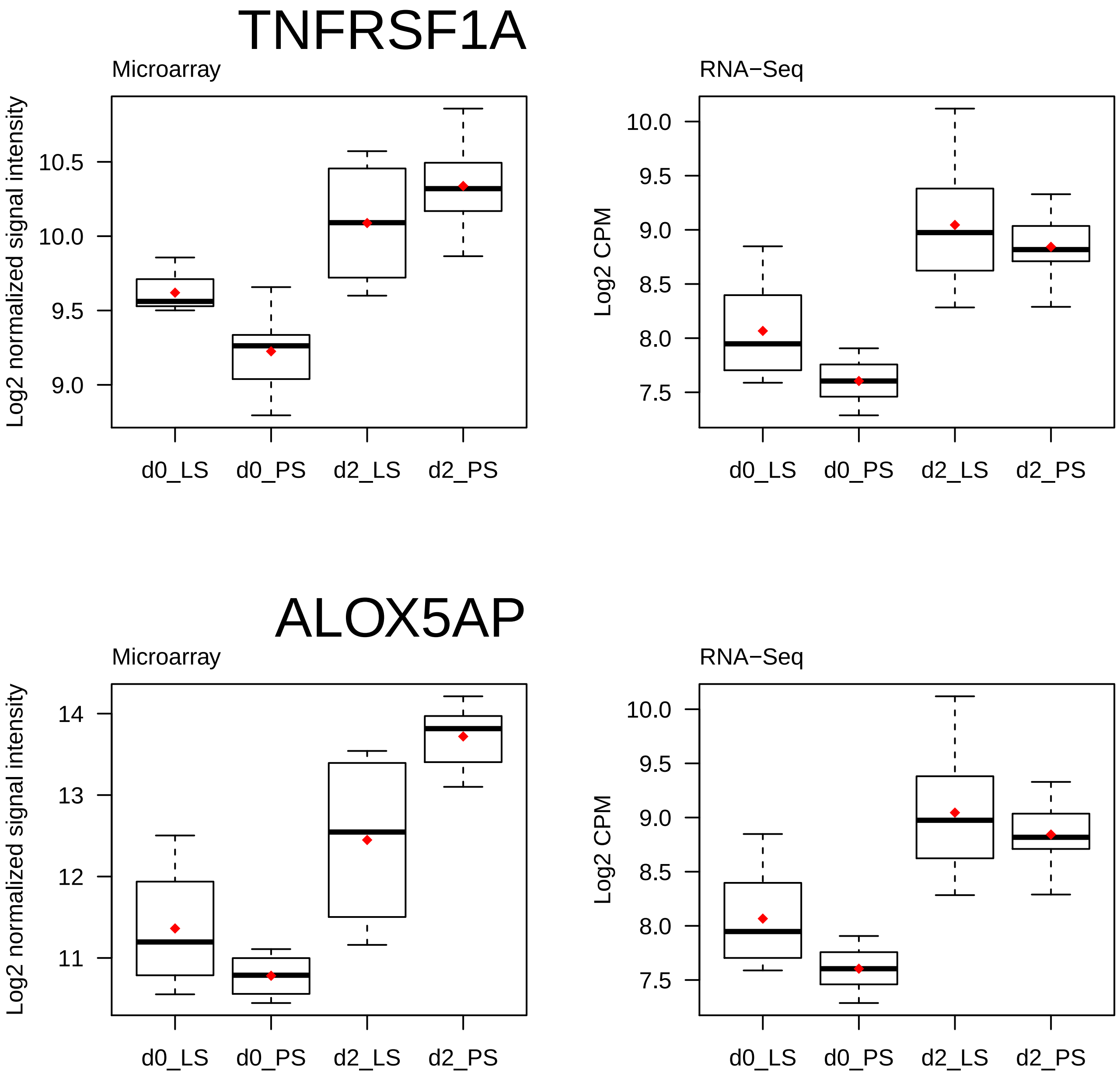

TNFRSF1A
Microarray
Log2 normalized signal intensity
9.0
9.5
10.0
10.5
d0_LS
d0_PS
d2_LS
d2_PS
RNA−Seq
Log2 CPM
7.5
8.0
8.5
9.0
9.5
10.0
d0_LS
d0_PS
d2_LS
d2_PS
ALOX5AP
Microarray
Log2 normalized signal intensity
11
12
13
14
d0_LS
d0_PS
d2_LS
d2_PS
RNA−Seq
Log2 CPM
7.5
8.0
8.5
9.0
9.5
10.0
d0_LS
d0_PS
d2_LS
d2_PS

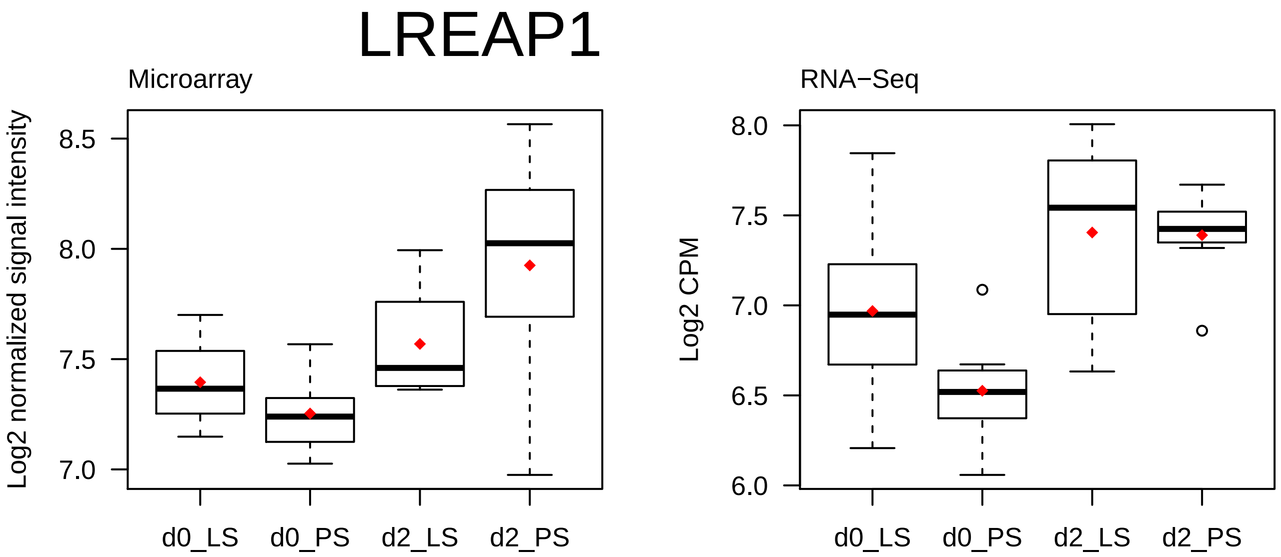
LREAP1
Microarray
Log2 normalized signal intensity
8.5
8.0
7.5
7.0
d0_LS
d0_PS
d2_LS
d2_PS
RNA−Seq
Log2 CPM
8.0
7.5
7.0
6.5
6.0
d0_LS
d0_PS
d2_LS
d2_PS

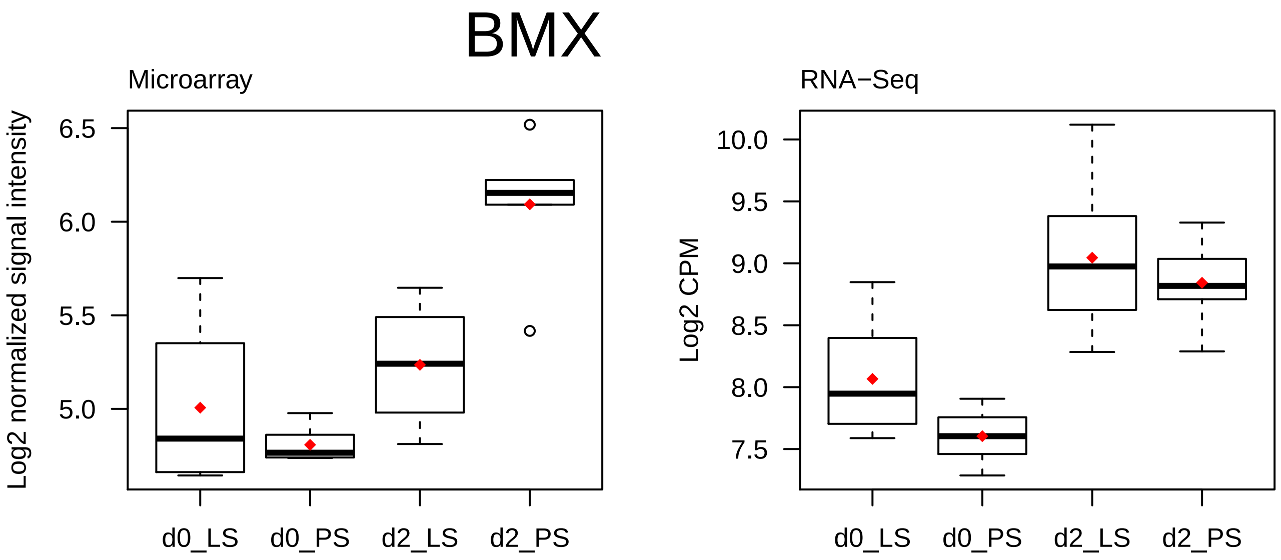
BMX
Microarray
Log2 normalized signal intensity
6.5
6.0
5.5
5.0
d0_LS
d0_PS
d2_LS
d2_PS
RNA−Seq
Log2 CPM
10.0
9.5
9.0
8.5
8.0
7.5
d0_LS
d0_PS
d2_LS
d2_PS

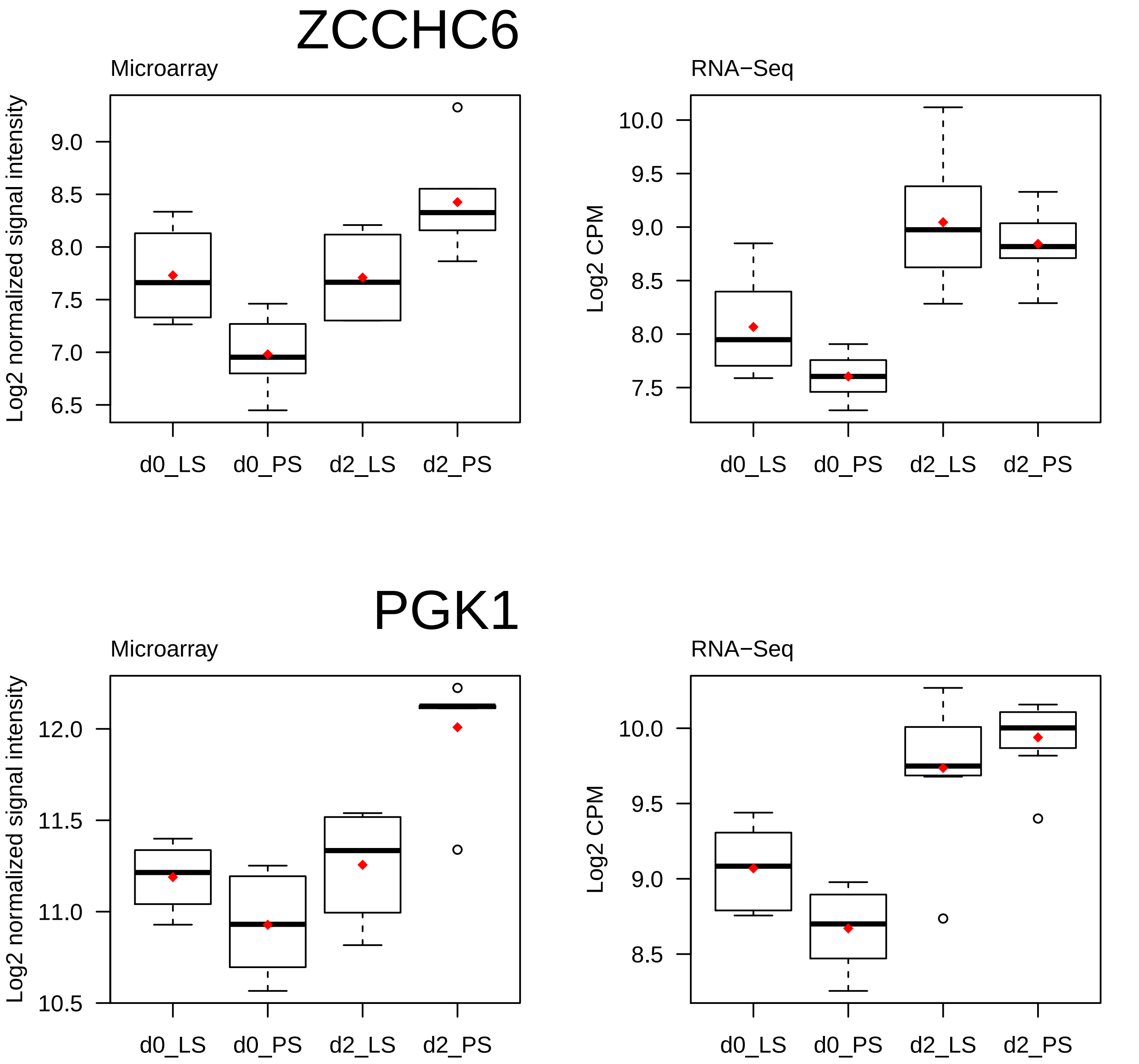

ZCCHC6
Microarray
Log2 normalized signal intensity
6.5
7.0
7.5
8.0
8.5
9.0
d0_LS
d0_PS
d2_LS
d2_PS
RNA−Seq
Log2 CPM
7.5
8.0
8.5
9.0
9.5
10.0
d0_LS
d0_PS
d2_LS
d2_PS
PGK1
Microarray
Log2 normalized signal intensity
10.5
11.0
11.5
12.0
d0_LS
d0_PS
d2_LS
d2_PS
RNA−Seq
Log2 CPM
8.5
9.0
9.5
10.0
d0_LS
d0_PS
d2_LS
d2_PS

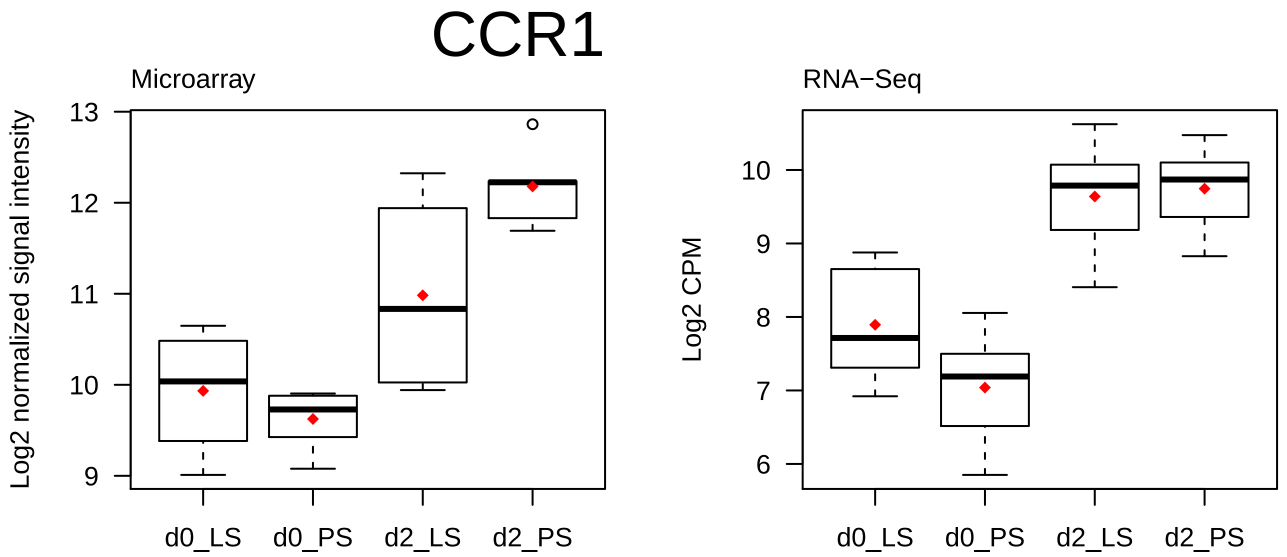
CCR1
Microarray
RNA−Seq
Log2 normalized signal intensity
Log2 CPM
9
10
11
12
13
6
7
8
9
10
d0_LS
d0_PS
d2_LS
d2_PS

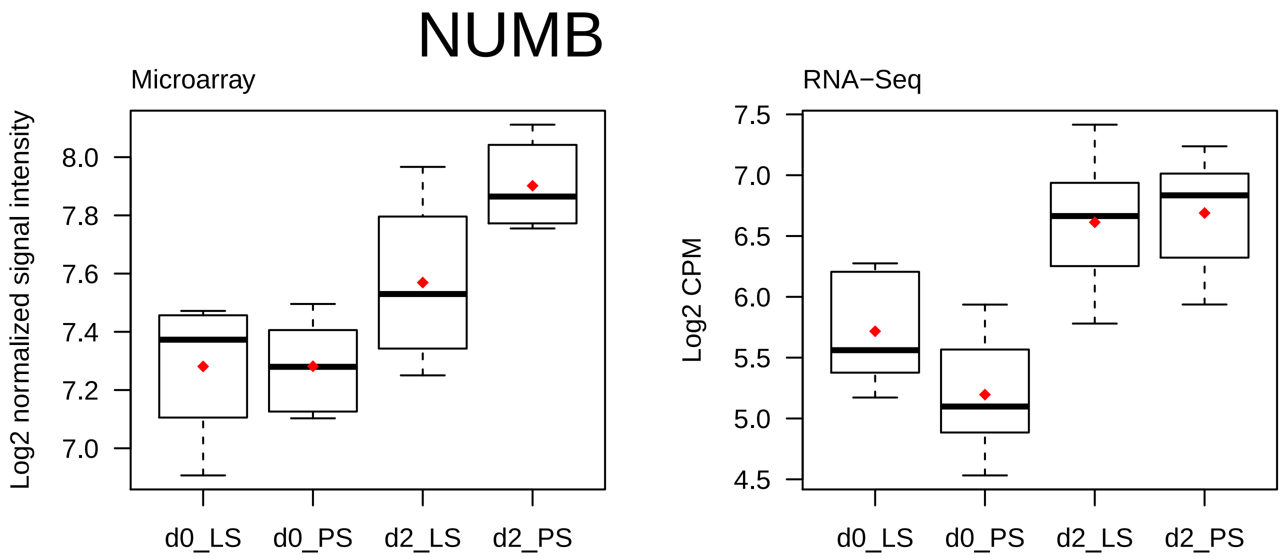
NUMB
Microarray
RNA−Seq
Log2 normalized signal intensity
Log2 CPM
7.0
7.2
7.4
7.6
7.8
8.0
4.5
5.0
5.5
6.0
6.5
7.0
7.5
d0_LS
d0_PS
d2_LS
d2_PS

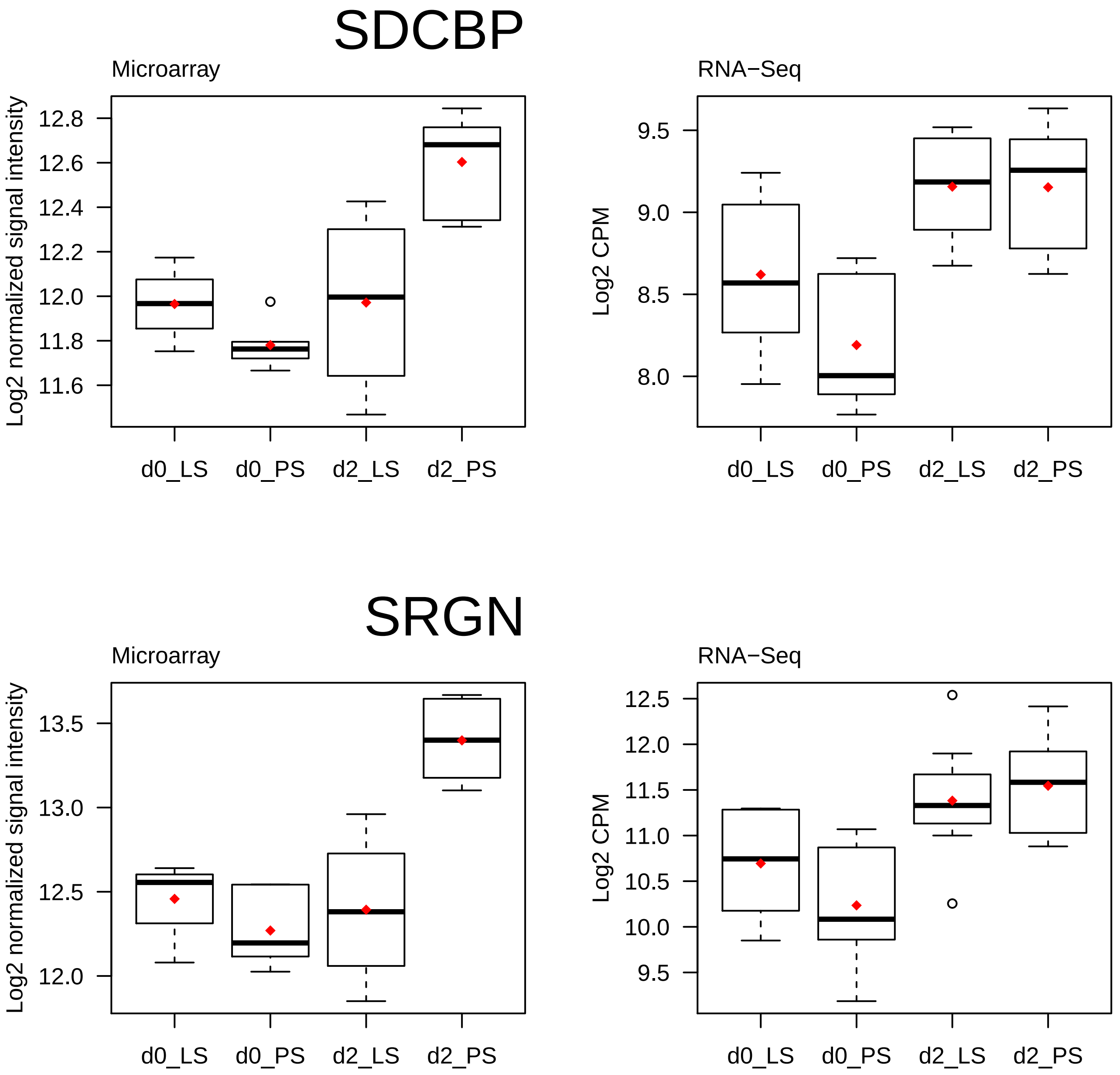
SDCBP
Microarray
Log2 normalized signal intensity
12.8
12.6
12.4
12.2
12.0
11.8
11.6
d0_LS
d0_PS
d2_LS
d2_PS
RNA−Seq
Log2 CPM
9.5
9.0
8.5
8.0
d0_LS
d0_PS
d2_LS
d2_PS
SRGN
Microarray
Log2 normalized signal intensity
13.5
13.0
12.5
12.0
d0_LS
d0_PS
d2_LS
d2_PS
RNA−Seq
Log2 CPM
12.5
12.0
11.5
11.0
10.5
10.0
9.5
d0_LS
d0_PS
d2_LS
d2_PS

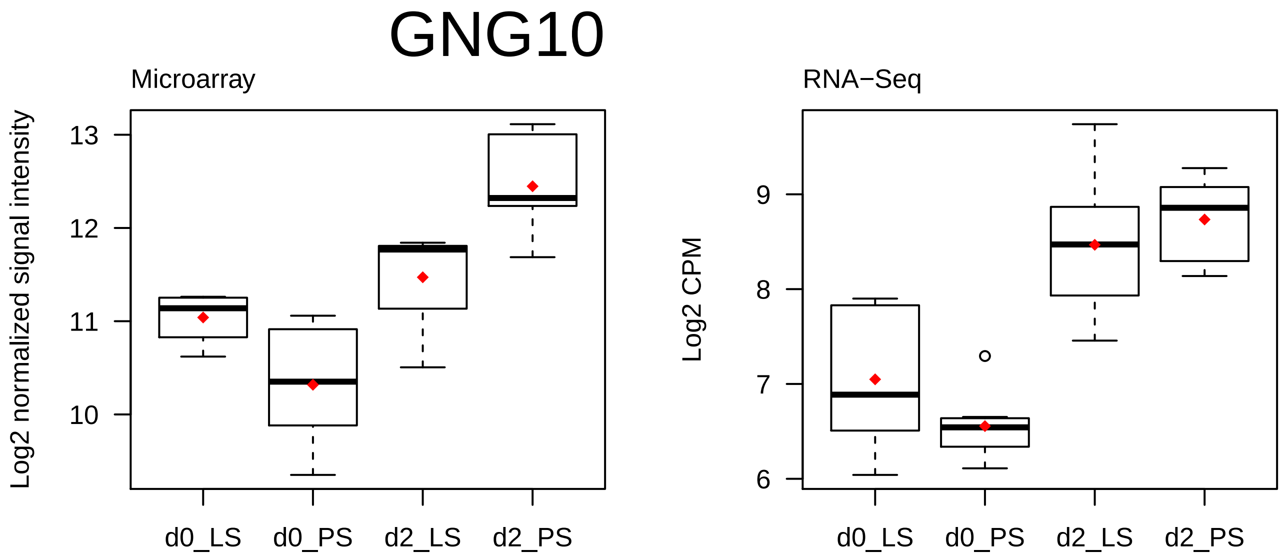
GNG10
Microarray
RNA−Seq
Log2 normalized signal intensity
Log2 CPM
13
12
11
10
9
8
7
6
d0_LS
d0_PS
d2_LS
d2_PS

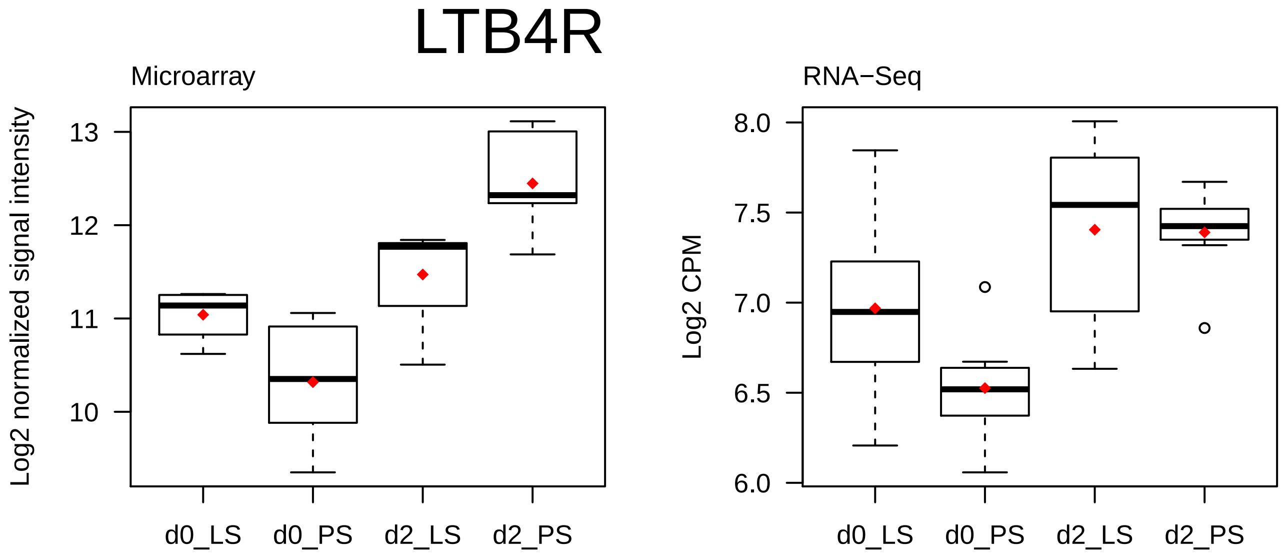
LTB4R
Microarray
RNA−Seq
Log2 normalized signal intensity
Log2 CPM
13
12
11
10
8.0
7.5
7.0
6.5
6.0
d0_LS
d0_PS
d2_LS
d2_PS

RNF149

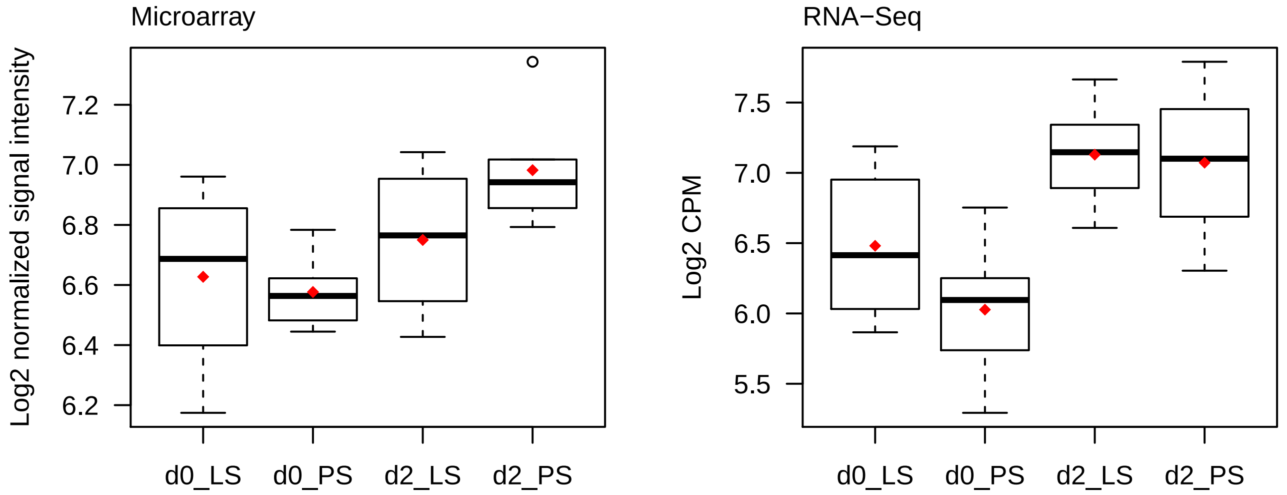

SLC11A1

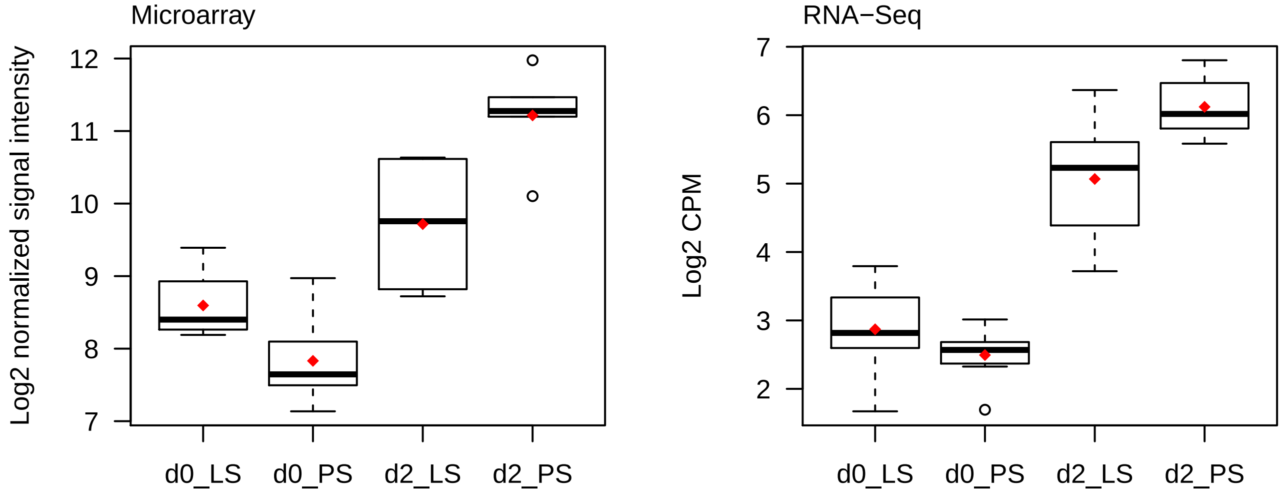

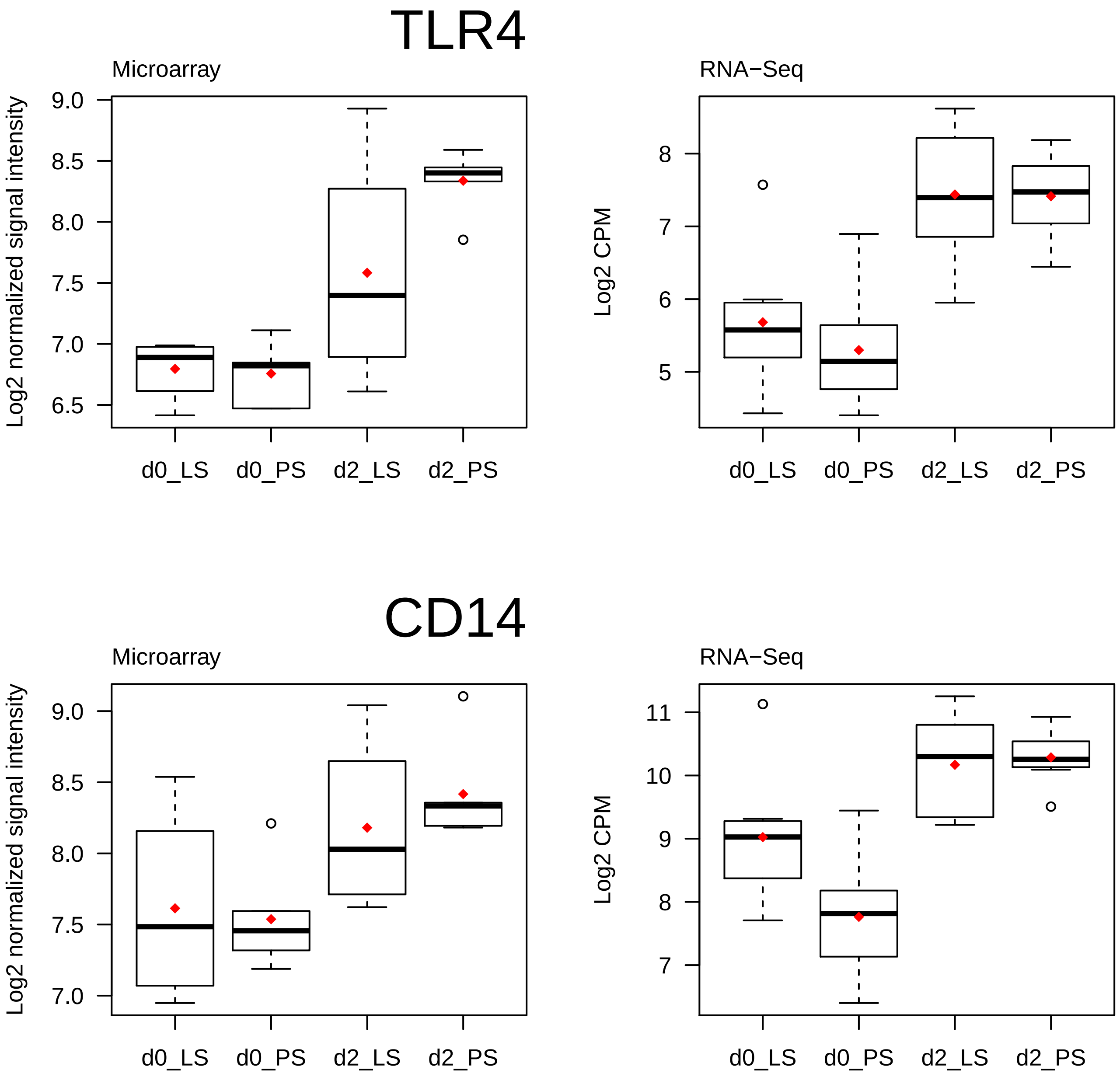
TLR4
Microarray
RNA−Seq
Log2 normalized signal intensity
Log2 CPM
9.0
8.5
8.0
7.5
7.0
6.5
8
7
6
5
d0_LS
d0_PS
d2_LS
d2_PS
CD14
Microarray
RNA−Seq
Log2 normalized signal intensity
Log2 CPM
9.0
8.5
8.0
7.5
7.0
11
10
9
8
7
d0_LS
d0_PS
d2_LS
d2_PS
